# Early evaluation of the effectiveness and cost-effectiveness of ctDNA-guided selection for adjuvant chemotherapy in stage II colon cancer

**DOI:** 10.1177/17588359241266164

**Published:** 2024-08-21

**Authors:** Astrid Kramer, Marjolein J. E. Greuter, Suzanna J. Schraa, Geraldine R. Vink, Jillian Phallen, Victor E. Velculescu, Gerrit A. Meijer, Daan van den Broek, Miriam Koopman, Jeanine M. L. Roodhart, Remond J. A. Fijneman, Valesca P. Retèl, Veerle M. H. Coupé

**Affiliations:** Department of Epidemiology and Data Science, Amsterdam University Medical Centers, De Boelelaan 1089a, Amsterdam, Noord-Holland 1081 HV, The Netherlands; Department of Epidemiology and Data Science, Amsterdam University Medical Centers, Amsterdam, The Netherlands; Department of Medical Oncology, University Medical Center Utrecht, Utrecht University, Utrecht, The Netherlands; Department of Medical Oncology, University Medical Center Utrecht, Utrecht University, Utrecht, The Netherlands; Department of Research and Development, IKNL, Utrecht, The Netherlands; The Sidney Kimmel Comprehensive Cancer Center, Johns Hopkins University School of Medicine, Baltimore, MD, USA; The Sidney Kimmel Comprehensive Cancer Center, Johns Hopkins University School of Medicine, Baltimore, MD, USA; Department of Pathology, Netherlands Cancer Institute, Amsterdam, The Netherlands; Department of Laboratory Medicine, Netherlands Cancer Institute, Amsterdam, The Netherlands; Department of Medical Oncology, University Medical Center Utrecht, Utrecht University, Utrecht, The Netherlands; Department of Medical Oncology, University Medical Center Utrecht, Utrecht University, Utrecht, The Netherlands; Department of Pathology, Netherlands Cancer Institute, Amsterdam, The Netherlands; Department of Psychosocial Research and Epidemiology, Netherlands Cancer Institute, Amsterdam, The Netherlands; Erasmus School of Health Policy and Management, Erasmus University Rotterdam, Rotterdam, The Netherlands; Department of Epidemiology and Data Science, Amsterdam University Medical Centers, Amsterdam, The Netherlands

**Keywords:** adjuvant chemotherapy, colon cancer, cost-effectiveness, ctDNA, prognostic biomarker

## Abstract

**Background:**

Current patient selection for adjuvant chemotherapy (ACT) after curative surgery for stage II colon cancer (CC) is suboptimal, causing overtreatment of high-risk patients and undertreatment of low-risk patients. Postoperative circulating tumor DNA (ctDNA) could improve patient selection for ACT.

**Objectives:**

We conducted an early model-based evaluation of the (cost-)effectiveness of ctDNA-guided selection for ACT in stage II CC in the Netherlands to assess the conditions for cost-effective implementation.

**Methods:**

A validated Markov model, simulating 1000 stage II CC patients from diagnosis to death, was supplemented with ctDNA data. Five ACT selection strategies were evaluated: the current guideline (pT4, pMMR), ctDNA-only, and three strategies that combined ctDNA status with pT4 and pMMR status in different ways. For each strategy, the costs, life years, quality-adjusted life years (QALYs), recurrences, and CC deaths were estimated. Sensitivity analyses were performed to assess the impact of the costs of ctDNA testing, strategy adherence, ctDNA as a predictive biomarker, and ctDNA test performance.

**Results:**

Model predictions showed that compared to current guidelines, the ctDNA-only strategy was less effective (+2.2% recurrences, −0.016 QALYs), while the combination strategies were more effective (−3.6% recurrences, +0.038 QALYs). The combination strategies were not cost-effective, since the incremental cost-effectiveness ratio was €67,413 per QALY, exceeding the willingness-to-pay threshold of €50,000 per QALY. Sensitivity analyses showed that the combination strategies would be cost-effective if the ctDNA test costs were lower than €1500, or if ctDNA status was predictive of treatment response, or if the ctDNA test performance improved substantially.

**Conclusion:**

Adding ctDNA to current high-risk clinicopathological features (pT4 and pMMR) can improve patient selection for ACT and can also potentially be cost-effective. Future studies should investigate the predictive value of post-surgery ctDNA status to accurately evaluate the cost-effectiveness of ctDNA testing for ACT decisions in stage II CC.

## Plain language summary

Effectiveness and cost-effectiveness of circulating tumour DNA-guided selection for adjuvant chemotherapy in patients with stage II colon cancer

Most patients with stage II colon cancer (CC) are cured by surgery. Therefore, guidelines recommend to only offer adjuvant chemotherapy to patients who have a tumor with high-risk features. However, current selection is suboptimal, leading to recurrence of cancer in 13% of low-risk patients and unnecessary administration of chemotherapy in some high-risk patients. Previous studies indicate that a biomarker, so-called circulating tumour DNA (ctDNA), could improve the selection of high-risk patients for adjuvant chemotherapy, as patients who have detectable ctDNA in their blood after surgery are likely to develop a recurrence. Despite its potential, implementation is still pending. Our study assessed the long-term effectiveness and costs associated with various ctDNA-guided strategies for selecting high-risk patients for adjuvant chemotherapy in stage II CC. We used an health-economic model to simulate a cohort of 1000 Dutch patients with stage II CC from diagnosis to death. Next, we compared the health outcomes and costs of the ctDNA-guided strategies to those when selection is based on the Dutch guideline. We found that a combination of the Dutch guideline and ctDNA was the most effective strategy, but not cost-effective. Additional analyses showed that ctDNA-guided selection were cost-effective if the costs of the ctDNA test were below 1500 euros, if the ctDNA test performed significantly better, or if patients with detectable ctDNA responded better to chemotherapy. Thus, while post-surgery ctDNA status is a good indicator for recurrence risk, specific criteria related to ctDNA test performance and costs, in addition to combining ctDNA with current high-risk features, should be met to achieve cost-effective implementation. Looking ahead, future studies should explore how patients with detectable ctDNA respond to chemotherapy for next assessments of the cost-effectiveness of ctDNA-guided strategies in selecting patients with stage II CC for adjuvant chemotherapy.

## Introduction

Colorectal cancer (CRC) is the second most common cause of cancer death in men and women worldwide. For localized disease, surgical resection is the recommended treatment.^1–3^ In stage II colon cancer (CC), approximately 80% of patients are already cured by the surgery alone, while ~20% of patients will develop a recurrence.^4–6^ Adjuvant chemotherapy (ACT) can reduce the recurrence risk, but the absolute survival benefit is small in stage II CC (<5% overall survival gain).^4,6^ ACT is therefore only recommended for high-risk stage II patients based on clinicopathological risk factors, but these criteria vary among countries.^7^

In the Netherlands, the two risk factors for patient selection for ACT in stage II CC are T-stage and mismatch repair (MMR) status.^3^ T-stage, as having a pT4 tumor is the primary clinicopathological risk factor for recurrence, and MMR status, as deficient mismatch repair (dMMR) tumors exhibit a lower recurrence risk compared to proficient miss match repair (pMMR) tumors.^2,3,8^ Therefore, the Dutch guideline recommends offering ACT to patients with a II pT4, pMMR tumor to reduce their recurrence risk.^3^ The current Dutch guideline is effective and cost-effective, and guideline adherence for ACT administration has improved since its last update in 2017.^9,10^ Nonetheless, these selection criteria are suboptimal, as the recurrence rate in low-risk patients is still substantial (12.5% in non-pT4 patients), and some high-risk patients who were cured by surgery receive unnecessary treatment, leading to futile toxicities and costs.^11^

Over the last decades, numerous potential biomarkers for CC prognosis have been reported in the literature.^12,13^ In addition, new innovations for biomarker detection have emerged, such as liquid biopsies, which is the concept of deriving information about the tumor from blood plasma or other body fluids. A promising biomarker measured in liquid biopsies is cell-free circulating tumor DNA (ctDNA), which are tumor-derived DNA fragments. Detecting ctDNA in blood allows for real-time assessment of tumor characteristics in a minimally invasive way. The presence of ctDNA after surgery indicates minimal residual disease and a high risk of recurrence.^14^ Multiple observational studies have suggested that ctDNA is a better marker for recurrence risk than current clinicopathological risk factors in stage II CC.^15–17^ Therefore, ctDNA testing can potentially improve the identification of high-risk patients for ACT. The first published randomized controlled trial (RCT) comparing ctDNA-guided referral for ACT to standard of care (i.e. ACT managed by the treating physician on a case-by-case basis, based on standard clinicopathological criteria) was performed in Australia.^18^ The publication showed that ACT referral based on ctDNA status was non-inferior to the current Australian standard of care, while fewer patients received ACT in the ctDNA-guided group (15% vs 28%).^18^ More RCTs evaluating the utility of ctDNA for ACT decisions after surgery are ongoing.^19^

One study has evaluated the cost-effectiveness of ctDNA-guided selection for ACT in stage II CC in the Australian setting.^20^ This economic evaluation suggests that ctDNA testing is potentially a cost-effective strategy (more effective, less costly). Since the criteria for ACT differ between countries, their conclusion on the cost-effectiveness of a ctDNA-guided strategy is specific to the Australian setting. To illustrate, a smaller proportion of stage II patients receives ACT in the Netherlands compared to Australia due to more narrow selection criteria (~9% vs ~28%), implicating less overtreatment in the Netherlands and impacting the cost-effectiveness.^18,21^

To evaluate the potential impact of ctDNA-guided ACT in a country with a more restrictive ACT administration, we performed an early model-based evaluation of the effectiveness and cost-effectiveness of ctDNA-guided selection strategies for ACT in stage II CC in the Netherlands. We first evaluated a strategy in which ACT selection was based on ctDNA status only. As studies have shown that ctDNA status in combination with clinicopathological risk factors could improve the stratification of high-risk patients, we also evaluated strategies combining ctDNA status with prognostic risk factors included in the Dutch guideline (pT4, pMMR).^15,18^ Finally, the impact of uncertain ctDNA-related parameters was explored in sensitivity analyses as well as the conditions for a cost-effective ctDNA-guided strategy in the Netherlands.

## Materials and methods

### The PATTERN model

**Figure 1.**
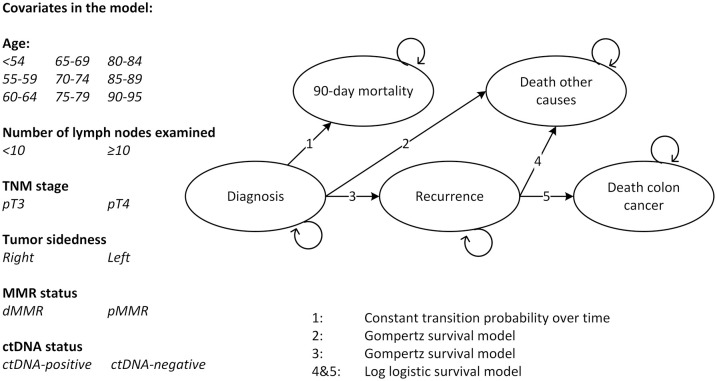
Structure of the PATTERN model. ctDNA, circulating tumor DNA; dMMR, deficient mismatch repair; pMMR, proficient mismatch repair.

The PATTERN model was developed earlier to evaluate the personalization of ACT in stage II CC. The PATTERN model has been internally and externally validated and has been described extensively elsewhere.^22^ The model parameters are reported in Supplemental Material 1. In brief, the PATTERN model is a Markov cohort model and describes the treatment and progression of stage II CC from diagnosis to death, taking into account the pathological-, clinical-, and biomarker features of the patient population (see Figure 1). The model has a lifelong time horizon, a 1-month cycle length, and has five health states: diagnosis, recurrence, 90-day mortality, death due to CC, and death due to other causes.

Data for model parametrization were derived from the Netherlands Cancer Registry (NCR; *n* = 2271 patients). It was assumed that all deaths within 90 days after diagnosis were due to surgical complications. For the other transitions in the model, parametric survival models including the relevant covariates (age, pT stage, tumor-sidedness, and evaluated lymph nodes) were used for the parametrization. These covariates were selected based on data availability, clinical relevance, and statistical significance as described in Jongeneel et al.^22^ Prognostic value of MMR status was derived from three external cohorts (*n* = 334 patients) and the treatment effect of ACT from pooled trial data.^23^ Both were incorporated into the transition from diagnosis to recurrence. The covariates included in the parametric survival models and MMR status were used to form subgroups with distinct characteristics in the simulated cohort. The subgroups were weighted such that the modeled cohort reflected the Dutch population with stage II CC. The reporting of this study conforms to the CHEERS guidelines (Supplemental Material 2).^24^

### Inclusion of ctDNA status

We supplemented the existing PATTERN model with ctDNA data, derived from the Molecular Early Detection of Colon Cancer (MEDOCC) study which was performed within the Prospective Dutch ColoRectal Cancer cohort (PLCRC).^25^ This observational study investigated the prognostic value of ctDNA status after surgery in 152 stage II and III CRC patients and used a tumor-informed targeted next-generation sequencing approach for ctDNA testing, meaning that both tumor and blood plasma were analyzed (unpublished data). From the PLCRC-MEDOCC study, a cohort of 86 stage II patients with data on post-surgery ctDNA status available who did not receive adjuvant chemotherapy was selected for the current study (See Table 1). This selection allowed us to estimate the prognostic value (i.e. HR) of ctDNA for recurrence without the effect of ACT but has the risk of resulting in a selective cohort of low-risk patients as high-risk patients are eligible for ACT. However, since clinical practice sometimes diverges from the clinical guidelines, the selected population also included patients who were considered to be at high risk (pT4, pMMR). The median time of blood collection after surgery was 11 days and the median follow-up time was 47 months. In 5/86 patients (5.8%) ctDNA presence was confirmed after surgery (i.e. had a ctDNA-positive test result) and 15/86 (17.4%) patients developed a recurrence. The HR for recurrence was 9.23 for ctDNA-positive versus ctDNA-negative patients. This information was incorporated into the PATTERN model, by assuming that also in the model 5.8% of patients were ctDNA positive after surgery and by adjusting the transition probability from diagnosis to recurrence (Figure 1, transition 3) with a HR of 9.23 for ctDNA-positive versus ctDNA-negative patients.

**Table 1.** Patient characteristics of the selected cohort from PLCRC-MEDOCC.

Variable	ctDNA positive patients	ctDNA negative patients	All patients
**All**	5	81	86
**Gender**			
**Male**	3 (60.0%)	53 (65.4%)	56 (65.1%)
**Female**	2 (40.0%)	28 (34.6%)	30 (34.9%)
**Age (average, SD)**	62.2 (10.9)	67.1 (10.2)	66.8 (10.2)
**T-stage**			
**pT3**	5 (100%)	74 (91.4%)	79 (91.9%)
**pT4**	0	7 (8.6%)	7 (8.1%)
**Mismatch** repair status			
**pMMR**	4 (80.0%)	62 (76.5%)	66 (76.7%)
**dMMR**	1 (20.0%)	17 (21.0%)	18 (20.9%)
**Missing**	0	2 (2.5%)	2 (2.3%)
**Lymph node yield** ^a^			
**<12**	2 (40.0%)	11 (13.6%)	13 (15.1%)
**⩾12**	3 (60.0%)	70 (86.4%)	73 (84.9%)
**Post-surgery ctDNA result**			
**Positive**	5	NA	5 (5.8%)
**Negative**	NA	81	81 (94.2%)
**Recurrence**			
**Yes**	4 (80.0%)	11 (13.6%)	15 (17.4%)
**No**	1 (20.0%)	70 (86.4%)	71 (82.6%)

a Note that the cutoff for dichotomization of the variable “lymph node yield” in this dataset (⩾12 lymph nodes) is different from the cutoff applied in the dataset used to parametrize the model (⩾10 lymph nodes). In this table, the variable “lymph node yield” is only used to describe the population on which the ctDNA-related parameters in the model are based. The variable was not used in further analyses.

ctDNA, circulating tumor DNA; dMMR, deficient mismatch repair; pMMR, proficient mismatch repair.

### Strategies

Six strategies were evaluated in this study for the identification of high-risk patients for ACT administration:

1. No ACT strategy: none of the patients receives ACT.
2. Current guideline strategy: patients with a pT4, pMMR tumor receive ACT.
3. ctDNA-only strategy: patients who have detectable ctDNA after surgery (i.e. ctDNA positive) receive ACT.
4. Combination strategy 1: patients with a pMMR tumor who are either ctDNA positive or have a pT4 tumor receive ACT.
5. Combination strategy 2: patients who have a pT4, pMMR tumor and all patients who have detectable ctDNA after surgery receive ACT.
6. Combination strategy 3: All patients who have a pT4 tumor and all patients who have detectable ctDNA after surgery receive ACT.

For all strategies, we assumed 100% adherence to the strategy. In the model, all high-risk patients were treated with 3 months of capecitabine plus oxaliplatin (CAPOX), as recommended by the Dutch guidelines.^3^ The treatment effect of CAPOX was assumed to be the same in all subgroups (HR 0.731).^23^

### Costs and health utilities

An overview of the costs and health utilities is shown in Table 2. Costs in the model were determined from a societal perspective. Cost for initial surgery, MMR testing, ACT, managing adverse events, absenteeism from work, patient’s travel to the hospital, surveillance, and treatment for recurrence of disease were already included in the model and updated to 2022 Euros.^3,26–33^ The costs for ctDNA testing were added and set to €2400, which was the rounded average of the tariffs for molecular diagnostics on liquid biopsy and tissue biopsy (billing codes 050544 and 050545) from 21 hospitals in the Netherlands (academic and non-academic).^34^

**Table 2.** Model inputs for costs and utilities.

Model inputs	Value	Proportion	References
**Costs**			
**Initial surgery** ^a^	€14,855		32
**Biomarker test**			
**MMR testing**	€72		32
**ctDNA testing**	€2400		34
**Treatment cost per full regimen**			
**CAPOX**^b^	€6692		3, 26, 30
**% early discontinuation of treatment**		0.25	27
**Adverse event cost per case** ^c^			
**Grade 3/4 neutropenia**	€111		26, 30, 35
**Febrile neutropenia**	€3885		26, 30, 35
**Grade 3/4 diarrhea**	€56		26, 30, 35
**Absenteeism costs per cycle** ^d^			30
**<55**	€6254		
**55–65**	€5800		
**Travel costs per cycle**	€10		30
**Surveillance costs** ^e^			
**Colonoscopy**	€997		3, 30
**Colonoscopy with complications**	€1264		31
**Ultrasound scan**	€97		3, 30
**CEA determination**	€9		3, 32
**Relapse costs**	€51,079		33
**Utilities**	No adjuvant treatment	Adjuvant treatment	
**Before surgery (month 1)**	0.85	0.85	25, 36
**After surgery/before chemotherapy (months** **2–3)**	0.85	0.81	25, 37
**During chemotherapy (month 4–6)**	0.86	0.83	25, 37
**First year after chemotherapy (month 7–18)**	0.86	0.83	25, 36
**More than 12 months after chemotherapy**	0.83	0.83	25, 36
**Recurrence (month**s **1–60 after recurrence)**	0.45	0.45	38–40

Source: Table adapted from Jongeneel et al.^41^ All costs were standardized to 2022 Euros, using the consumer price index.^26^

a DBC tariffs from 24 hospitals in the Netherlands were averaged.

b The treatment with CAPOX consisted of four cycles of 3 weeks.^3^

c Costs were based on adverse event rates and follow-up care per adverse event category. For neutropenia follow-up, care was defined as a visit to the outpatient clinic, for febrile neutropenia as a hospital stay of 5 days, and for diarrhea as oral rehydration medication.^26,30^

d To calculate the absenteeism costs, we assumed that (i) the male-to-female ratio was 0.47/0.53^42^; (ii) number of hours worked per week was 40 and 38 for men and 28 and 25 for women in the age groups <55 and 55–65, respectively; and (iii) patients do not work during chemotherapy.^26^ The absenteeism costs were calculated according to the friction cost approach.

e Surveillance costs were calculated according to the Dutch guideline for colon cancer surveillance.

CAPOX, capecitabine/oxaliplatin; CEA, carcinoembryonic antigen; MMR, mismatch repair.

Health utilities were based on patient-level data of 859 Dutch patients included in the PLCRC study, who were diagnosed with stage II or III CC between 2011 and 2019 and who completed the EQ-5D-5L.^36^ The scores from the EQ-5D-5L were summarized into utility scores using the Dutch tariff.^43^ Costs were discounted by 4% and health effects by 1.5%, according to the Dutch guidelines for economic evaluations.^30^

### Outcomes

Model outcomes for each strategy were the number of recurrences and CC deaths per 1000 patients, and the number of life years, quality-adjusted life years (QALYs), and total lifetime costs per patient. The net monetary benefit (NMB) was calculated as total QALYs * willingness-to-pay threshold − total costs. The willingness-to-pay (WTP) threshold was set to €50,000 per QALY. In addition, an incremental analysis was done and cost-effectiveness ratios (ICERs) were calculated for consecutive non-dominated strategies by dividing the difference in costs by the difference in QALYs resulting in the costs per QALY gained. Strategies were considered dominant and were eliminated from further analysis if an alternative strategy had lower costs and equal or higher effectiveness. Strategies that had a higher ICER than a more effective strategy were also eliminated, by extended dominance. A strategy was considered cost-effective when the ICER did not exceed the WTP threshold of €50,000 per QALY.^30^

### Sensitivity analyses

Sensitivity analyses were performed to evaluate the impact of ctDNA-related parameters with a high degree of uncertainty. An overview of the new model inputs in each sensitivity analysis is shown in Supplemental Material 3.

In the first sensitivity analysis, the costs of ctDNA testing were lowered as the costs for sequencing are expected to decrease. The costs per ctDNA test varied between €1000 and €2000 with steps of €250.

In the second sensitivity analysis, the impact of lower strategy adherence was evaluated, because guidelines are not fully adhered to in daily clinical practice. For each strategy, it was assumed that among patients that are recommended to receive ACT, only 44% of patients <75 years of age received ACT, and 11% of patients ⩾75 years. This is consistent with data from the NCR in 2018–2019.^9,41^

In the third sensitivity analysis, the treatment effect of ACT was adjusted such that patients with detectable ctDNA after surgery responded better to ACT than patients without detectable ctDNA, as was recently reported for high-risk stage II and stage III patients in a publication from the GALAXY study (i.e. observational arm of the ongoing CIRCULATE-Japan study).^16^ In the base-case analysis, the treatment effect of ACT on recurrence risk was estimated as an HR of 0.731 for all patients. In this sensitivity analysis, the base-case treatment effect HR of 0.731 was reduced in ctDNA-positive patients with 0.2 and 0.4 to HRs of 0.531 and 0.331, respectively. The HRs for treatment effect for ctDNA-negative patients were calibrated to maintain the same average treatment effect in the whole cohort (resulting in HRs of 0.775 and 0.839, respectively).

Finally, there is currently a wide variation in ctDNA methods and panels for the analysis of post-surgery ctDNA in CC, leading to substantial variability in test performance.^14^ In addition, ctDNA technologies may improve and reach a higher sensitivity in the future, due to the rapid developments in the field. Therefore, we evaluated a scenario in which we assumed increased performance of the ctDNA test, leading to a higher positivity rate of ctDNA test results (8%, 10%, and 12% of patients have a ctDNA-positive result instead of the 5.8% in the base-case analysis). We assumed that the recurrence risk in the ctDNA-positive population remained the same as in the base-case analysis. Providing that the size of the ctDNA-positive population increases in this scenario, and that their recurrence risk remains the same, the recurrence risk in ctDNA-negative patients required recalibration to ensure the total number of recurrences in the cohort remained the same (see Supplemental Material 3).

## Results

### Base-case analysis

#### Effectiveness

The model outcomes of the six strategies are shown in Table 3 and Supplemental Material 4. The model predicted 163 recurrences and 138 CC deaths in the lifetime of 1000 patients if none of the patients received ACT. All ACT strategies were more effective and resulted in fewer recurrences and CC deaths. The current guideline strategy resulted in 155 recurrences and 131 CC deaths. Compared to the current guideline, the ctDNA-only strategy was less effective (158 recurrences and 134 CC deaths) and the combination strategies were more effective. Overall, combination strategy 3 (pT4 patients and ctDNA-positive patients) was the most effective selection strategy, resulting in the lowest number of recurrences and CC deaths (149 recurrences, 126 CC deaths). Note that in this strategy the highest proportion of patients received ACT.

**Table 3.** Results of the base-case analysis.

Strategy	% received ACT^a^	Recurrences^b^	CC deaths^b^	QALYs^c^	Costs^c^	NMB^d^	ICER
No ACT	0.00%	163	138	8.027	€26,227	€375,131	Reference
Current guideline (pT4, pMMR)	10.98%	155	131	8.079	€26,911	€377,0333	€13,223
ctDNA only (ctDNA positive)	5.81%	158	134	8.063	€28,735	€374,392	Dominated
Combination 1 (pT4, pMMR and ctDNA positive, pMMR)	15.24%	151	127	8.108	€28,970	€376,440	Dominated^e^
Combination 2 (pT4, pMMR, and ctDNA positive)	16.16%	150	127	8.113	€29,402	€376,244	Dominated^e^
Combination 3 (pT4 and ctDNA positive)	18.10%	149	126	8.117	€29,481	€376,370	€67,413

Explanation of the dominated ICERs: The strategy ctDNA only was considered dominated as it resulted in less QALYs and more costs than the Current Guideline strategy. Combinations 1 and 2 were dominated through extended dominance by Combination 3 as their ICERs were higher.

a Proportion of that cohort that received adjuvant chemotherapy.

b Total number in a simulated cohort of 1000 patients.

c Mean per patient.

d Based on a willingness-to-pay threshold of €50,000/QALY.

e Through extended dominance.

ACT, adjuvant chemotherapy; CC, colon cancer; ctDNA, circulating tumor DNA; ICER, incremental cost-effectiveness ratio; NMB, net monetary benefit; pMMR, proficient mismatch repair; QALYs, quality-adjusted life years.

#### Cost-effectiveness

**Figure 2.**
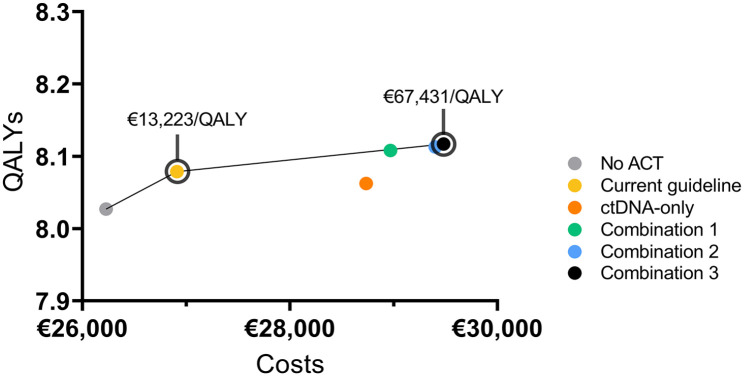
Cost-effectiveness plane with base-case results. Cost-effectiveness plane depicting the average discounted costs in € and quality-adjusted life-years (QALYs) of each strategy per patient. The black line represents the cost-effectiveness frontier. Note that the ctDNA-only strategy and combination strategies 1 and 2 are not on the cost-effectiveness frontier. Current guideline: pT4, pMMR patients; ctDNA-only: ctDNA positive patients; Combination 1: pT4, pMMR patients and ctDNA positive, pMMR patients; Combination 2: pT4, pMMR patients and ctDNA-positive patients; Combination 3: pT4 patients and ctDNA-positive patients.

The results from the base-case analysis are shown in Table 3 and are displayed on a cost-effectiveness plane depicting the costs and QALYs of each strategy (Figure 2). The No ACT strategy resulted in the lowest number of QALYs and costs, and combination strategy 3 (pT4 and ctDNA-positive) in the highest number of QALYs and costs.

The selection strategies on the cost-effectiveness frontier were the current guideline strategy and combination strategy 3 (pT4 and ctDNA positive) with respective incremental ICERs of €13,223 and €67,431 per QALY. The ctDNA-only strategy was dominant as it resulted in higher costs and less QALYs than the current guideline. Combination strategies 1 and 2 were dominated through extended dominance. Combination 3 was not cost-effective, because the ICER exceeded the WTP threshold of €50,000 per QALY. The ICER of the current guideline strategy was below the WTP threshold, and therefore the preferred strategy.

### Sensitivity analyses

**Figure 3.**
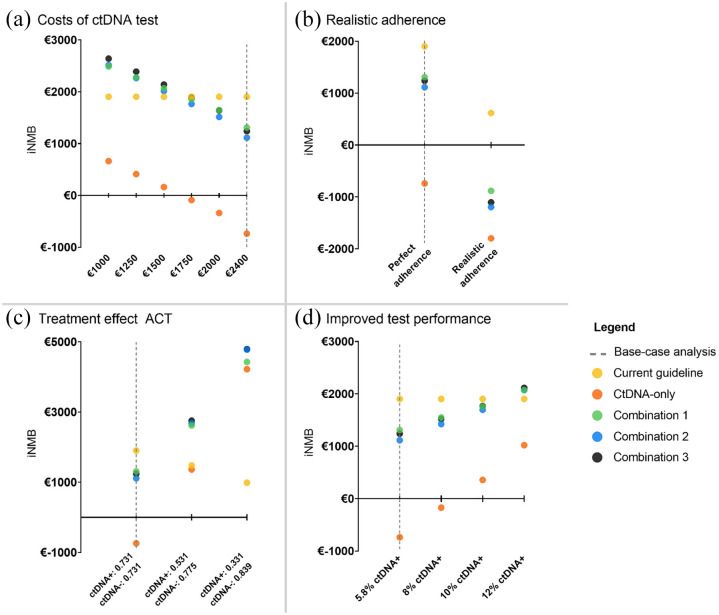
Results of the sensitivity analyses in terms of iNMB compared to the no ACT strategy. (a) Varying the costs of ctDNA testing. The *X*-axis depicts the costs per test per patient. (b) Strategy adherence. The *X*-axis depicts the adherence to the strategy. (c) Varying the treatment effect of ACT. The *X*-axis depicts three assumptions for the treatment effect of ACT for ctDNA-positive patients and ctDNA-negative patients. (d) Varying ctDNA test performance. The *X*-axis depicts the percentage of patients with a ctDNA-positive test result. Note the different scales used for the *Y*-axes in Figures a–d. Current guideline, pT4, pMMR patients; ctDNA-only, ctDNA positive patients; Combination 1, pT4, pMMR patients and ctDNA positive, pMMR patients; Combination 2, pT4, pMMR patients and ctDNA-positive patients; Combination 3, pT4 patients and ctDNA-positive patients; iNMB, incremental net monetary benefit.

The results of the sensitivity analyses are shown in Supplemental Materials 5–9 and are presented in Figure 3 in terms of incremental NMB (iNMB) compared to No ACT. The strategy that has the highest iNMB is considered the most favorable strategy in terms of cost-effectiveness at a WTP threshold of €50,000/QALY.

The results of the sensitivity analyses varying the costs of ctDNA testing showed that if the costs of ctDNA testing were higher than €1500 per test, the current guideline strategy had the highest iNMB and was, therefore, the preferred strategy (Figure 3(a); Supplemental Material 5). At costs of ctDNA testing lower than €1500, the iNMBs of the combination strategies became higher than the current guideline strategy, and thus more favorable than the current guideline in terms of cost-effectiveness.

The results of the sensitivity analysis changing the strategy adherence to more realistic adherence showed decreased NMB for all strategies (Figure 3(b); Supplemental Material 6). However, given the same adherence pattern per strategy, the order of the strategies remained the same.

The results of the third sensitivity analysis showed that if the effectiveness of ACT was increased (in two steps) for ctDNA-positive patients, while the treatment effectiveness was decreased for ctDNA-negative patients, more recurrences were prevented in the ctDNA-only strategy and the combination strategies than in the base-case analysis (Figure 3(c); Supplemental Material 7). In contrast to the base-case analysis, the ctDNA-only strategy was more effective than the current guideline if the treatment effect of ACT would have an HR of 0.531 for recurrence in ctDNA-positive patients (153 and 156 recurrences in ctDNA-only vs current guideline strategy, respectively). However, the ctDNA-only strategy still had lower NMB than the current guideline strategy. Combination strategies had higher NMBs, with combination 3 having the highest NMB and an ICER of €39,222 per QALY. Under the assumption that the treatment effect of ACT would have an HR of 0.331 for recurrence in ctDNA-positive patients, the benefits of all ctDNA strategies compared to current guidelines further improved and their NMBs for all ctDNA strategies were substantially higher than for the current guideline strategy. The NMB of the ctDNA-only strategy was close to the NMBs of the combination strategies while treating the smallest proportion of patients (5.8% vs 15.2%–18.0%).

The results of the sensitivity analysis simulating the improved performance of the ctDNA test are shown in Figure 3(d) and Supplemental Material 8. The results show that in the analyses in which 8%, 10%, and 12% of patients were identified as ctDNA positive, fewer recurrences occurred in the low-risk groups and more recurrences occurred in the high-risk groups in the ctDNA-only and combination strategies (Supplemental Materials 8B and 9). This indicates that in these sensitivity analyses, ctDNA testing improved the patient selection. However, only if the test identified at least 10% of patients as ctDNA positive, the ctDNA-only strategy was more effective than the current guideline strategy. In terms of cost-effectiveness, the current guideline strategy remained the most favorable strategy under the assumption that the test identified 8% or 10% of patients as ctDNA positive (Figure 3(d)). Under the assumption that the test identified 12% of patients as ctDNA positive, the NMBs of the combination strategies were higher than the current guideline and therefore were more favorable (ICER of combination 3 strategy: €46,342/QALY; Supplemental Material 8A).

## Discussion

In this study, we performed an early model-based evaluation of the effectiveness and cost-effectiveness of ctDNA-guided selection strategies for ACT in stage II CC. Our results suggest that selecting patients for ACT based on ctDNA status only is less effective than selection conforms to the current Dutch guidelines (pT4, pMMR). Combining ctDNA status with pT4 or with both pT4 and pMMR status was more effective than the current guideline. The best combination strategy, ACT in patients with either a pT4 tumor or a post-surgery ctDNA-positive test, was not cost-effective compared to the current guideline as the ICER exceeded the WTP threshold of €50,000 per QALY. However, this conclusion was sensitive to assumptions concerning ctDNA-related parameters in the model.

Although the combination strategies were not cost-effective in the base-case analysis, they have the potential to be cost-effective, primarily a combination strategy of ctDNA status and pT4. Given all assumptions and keeping all other parameters constant, selecting ctDNA-positive and pT4 patients for ACT was cost-effective if the costs of ctDNA testing were lower than €1500 per patient, or if ctDNA-positive patients respond substantially better to ACT than ctDNA-negative patients, or if the ctDNA test performance improves substantially. The sensitivity analysis for strategy adherence shows that the selection strategies including ctDNA can only be successful in case the strategy adherence is high.

For those ctDNA-guided strategies that are cost-effective in the sensitivity analyses, ICERs fell within the range of €30.000–€50.000 per QALY. This means that for WTP thresholds substantially lower than €50.000/QALY, as, for example, in countries like the United Kingdom and Spain, ctDNA-guided strategies are less likely to be cost-effective than with the current WTP threshold. To illustrate, if the WTP threshold would be half of the current threshold, that is, €25.000/QALY instead of €50.000/QALY, the only ctDNA-guided strategy that will remain cost-effective is the ctDNA-only strategy in the sensitivity analysis in which ctDNA status is strongly predictive of treatment response. This implies that in countries with lower WTP thresholds, ctDNA testing would need to meet even stricter conditions for cost-effective implementation. However, note that our results cannot be directly translated to other countries, as recurrence rates, survival, and cost estimates in this study are specific to the Dutch setting, and the clinical guideline for ACT differs among countries. For the interpretation of our results, two major aspects of the ctDNA data incorporated into the health economic model should also be carefully considered: the patient population in our dataset and the ctDNA test. First of all, our ctDNA data were derived from an observational study and based on a small, very selected group of stage II CC patients who did not receive ACT (*n* = 86), allowing estimation of the prognostic value for recurrence for ctDNA-positive versus ctDNA-negative patients without the effect of ACT. However, patients who do not receive ACT generally have a lower recurrence risk, which is also shown in the slightly lower proportion of patients with a pT4 tumor compared to previously reported proportions of pT4 tumors in Dutch stage II CC patients (8.1% vs 9%–13%).^8,22^ This may have impacted the ctDNA positivity rate (5.8% ctDNA-positive post-surgery) and the prognostic value of ctDNA testing (HR of 9.23 recurrence in ctDNA-positive vs ctDNA-negative patients) incorporated into our model and therefore also our model predictions. Second, our results are based on a single test with specific performance characteristics, while various ctDNA technologies for the detection of minimal residual disease exist with different methods, test performances, and prices. The choice of test therefore also impacts the model predictions.

Direct comparisons of our ctDNA data to other studies are complicated due to the large heterogeneity in studies investigating the utility of ctDNA testing to guide ACT decisions in CRC. Variations in many logistical aspects of ctDNA testing (e.g. timing of sample collection and method of analysis) and study population (e.g. stage of disease and received treatment) contribute to this heterogeneity.^14^ Faulkner et al. reported that this heterogeneity resulted in wide ranges in positivity rate (7.9%–35.6%) and prognostic value (HR 1.46–20 in univariate analyses, HR 1.55–14 in multivariate analyses) in their meta-analysis, including studies with all cancer stages and ctDNA methods. Our prognostic value of ctDNA status (HR 9.23) falls within the reported range, but our lower positivity rate (5.8% ctDNA positive) can partially be explained by the fact that this meta-analysis also included stage III and resectable stage IV patients. Studies focusing on or stratifying for stage II patients narrow the range in the positivity rate (7.9%–15.4%).^15,16,18^ Both our low-risk population and the different ctDNA testing methods can probably explain the lower positivity rate than reported in other studies for stage II CC. Even though the ctDNA test in this study might be outperformed by newly developed ctDNA tests, our results show that its addition to clinicopathological factors can still improve the selection of high-risk patients for ACT.

Given the rapidly evolving field of ctDNA testing and its continuous advancements aimed at improving ctDNA test performance, it is interesting how test performance affects the cost-effectiveness of various ACT strategies. Our sensitivity analysis showed that a substantial increase in test performance was necessary for the ctDNA-only strategy to be more effective than the current guideline (at least 10% of patients identified as ctDNA-positive). In addition, only in the analysis in which the test identified 12% of patients as ctDNA positive, the combination strategy of ctDNA and pT4 was cost-effective. This implies that a substantial improvement in test performance is needed for a combination strategy to become cost-effective in the Netherlands.

In another sensitivity analysis, we simulated that ctDNA is both a predictive biomarker and a prognostic biomarker, assuming a substantially higher treatment effect of ACT in ctDNA-positive patients compared to ctDNA-negative patients. This assumption greatly influenced cost-effectiveness, favoring the combination strategies over the current guideline. The combination of ctDNA status and pT4 emerged as the most favorable strategy in our extreme sensitivity analysis. However, the ctDNA-only strategy is also worth considering, given its lower proportion of treated patients, with similar health effects. Current evidence on the predictive value of ctDNA testing is limited, with a study by Kotani et al. suggesting higher ACT effectiveness in ctDNA-positive patients in high-risk stage II and stage III CRC (HR 0.152, *p* < 0.0001) compared to ctDNA-negative patients (HR for disease-free survival of 0.585 at 18 months, *p* = 0.16).^16^ These results should be interpreted with caution since the follow-up time was short and the sample size was limited. Ongoing clinical trials will provide more evidence on the predictive value of ctDNA status for ACT in localized CC.^19,44^

The study of To et al. also assessed the cost-effectiveness of ctDNA testing for ACT decisions in stage II CC. In contrast to our results, they found that a ctDNA-guided strategy (i.e. ctDNA-only) is likely to be cost-effective (more effective, less costly) in Australia, with fewer recurrences and small gains in QALYs.^20^ This reduction in recurrences as well as the reduction in chemotherapy delivery (ctDNA-guided strategy: 8.69% vs standard of care: 22.6%) drove the lower costs in the ctDNA-guided strategy in their study. In our study, the ctDNA-only strategy also reduced chemotherapy delivery (ctDNA-only strategy: 5.8% vs current guideline: 10.98%), but resulted in more recurrences, making the ctDNA-only strategy more costly and less effective than the current guideline. There are many differences between the two studies that could explain the difference in results. Besides the difference in current ACT administration, another important difference between the studies lies in the ctDNA data. The ctDNA data of To et al. was derived from a previous publication, in which the recurrence rate in the ctDNA-positive group was similar to our study. However, the recurrence rate in the ctDNA-negative group was lower (To: 9.8%, our study: 13%), which is reflected in the substantial difference in prognostic HRs (To: HR 18, our study: HR 9.23). This could partially explain the discrepancy between the studies.

A strength of this study is the use of the internally and externally validated PATTERN model, which can therefore be considered a reliable model for accurately simulating treatment and disease progression from diagnosis to death in the Dutch stage II CC population.^22^ Assumptions for the ctDNA parameters were necessary due to evidence gaps in the ctDNA literature and the limited sample size of our dataset which was derived from an observational study. These main assumptions were explored through sensitivity analysis. Besides these assumptions, we were required to assume that the positivity rate and prognostic value of ctDNA were uniform across all subgroups, given the current lack of evidence associating ctDNA status with other risk factors in stage II CC patients. Probabilistic sensitivity analysis was not conducted due to the inability to include ctDNA-related parameters in the random sampling procedures, as varying specific ctDNA parameters (e.g. positivity rate of ctDNA test) required calibration of other linked ctDNA parameters (e.g. recurrence risk in ctDNA-negative patients) leading to a prohibitive computation time-intensive procedure. Although a probabilistic sensitivity analysis would have generated valuable information, our sensitivity analyses showed how the key parameters influenced the results. The ongoing RCTs will provide more evidence of the key uncertainties. For cost-effective implementation of ctDNA testing in clinical practice in the Netherlands, a sensitive assay is required, but also costs of the test should be restricted. A more favorable and likely development is an improvement of the ctDNA test performance, reduction in costs, and increase in strategy adherence. Finally, serial testing in ctDNA-negative patients has also been suggested in the literature to reduce undertreatment in ctDNA-negative patients.^18,45^ When the added value of serial testing has been shown, the cost-effectiveness of this strategy should also be assessed since serial testing is also associated with a higher financial burden.

## Conclusion

Our results showed that adding ctDNA testing to current clinicopathological selection criteria for adjuvant chemotherapy can improve the selection of patients for ACT in stage II CC in the Netherlands in terms of CC recurrence rate and survival, although this is not (yet) cost-effective. However, considering the fast developments in ctDNA technologies and the expected reduction in sequencing costs, our results also show that ctDNA selection strategies have the potential to become cost-effective, especially when combined with pT4. Most importantly, it is crucial to investigate whether ctDNA status is predictive of ACT response.

## Supplemental Material

sj-docx-1-tam-10.1177_17588359241266164 – Supplemental material for Early evaluation of the effectiveness and cost-effectiveness of ctDNA-guided selection for adjuvant chemotherapy in stage II colon cancer Supplemental material, sj-docx-1-tam-10.1177_17588359241266164 for Early evaluation of the effectiveness and cost-effectiveness of ctDNA-guided selection for adjuvant chemotherapy in stage II colon cancer by Astrid Kramer, Marjolein J. E. Greuter, Suzanna J. Schraa, Geraldine R. Vink, Jillian Phallen, Victor E. Velculescu, Gerrit A. Meijer, Daan van den Broek, Miriam Koopman, Jeanine M. L. Roodhart, Remond J. A. Fijneman, Valesca P. Retèl and Veerle M. H. Coupé in Therapeutic Advances in Medical Oncology

## Declarations

**Ethics approval and consent to participate:** Ethical approval was not required for this study. This modeling study did not include any human participants or animals. According to the Central Committee on Research involving Human Subjects (CCMO, The Hague, Netherlands), this study type does not require ethics approval in The Netherlands.

**Consent for publication:** Not applicable.

**Availability of data and materials:** The data from the PATTERN model that support the findings of this study are available from the Decision Modeling Center, which is part of the Amsterdam University Medical Center, location VUmc upon reasonable request. Restrictions apply to the availability of data from the PLCRC-MEDOCC study, which were used with the permission of the principal investigator (G. A. Meijer).
