# Supplementary material for: Early evaluation of the effectiveness and cost-effectiveness of ctDNA-guided selection for adjuvant chemotherapy in stage II colon cancer
Source: Ther Adv Med Oncol. 2024 Aug 21;16:17588359241266164. doi: 10.1177/17588359241266164 (PMC11339739; doi:10.1177/17588359241266164)
Supplement: sj-docx-1-tam-10.1177_17588359241266164 – Supplemental material for Early evaluation of the effectiveness and cost-effectiveness of ctDNA-guided selection for adjuvant chemotherapy in stage II colon cancer [file sj-docx-1-tam-10.1177_17588359241266164.docx]

**Supplementary Materials**

# Supplementary Materials 1. Parameter estimates specifying transitions in the PATTERN model.

|  | **Transition Diagnosis to 90DM**  **(DIAG-90DM)** | **Transition Diagnosis to DOC**  **(DIAG-DOC)** | | **Transition Diagnosis to recurrence**  **(DIAG-REC)** | | **Transition Recurrence to Death**  **(REC-DOC + REC-DCC)^a^** | | **Transition Recurrence to DOC**  **(REC-DOC)** |
| --- | --- | --- | --- | --- | --- | --- | --- | --- |
| Parametric distribution | NA | Gompertz | | Gompertz | | Log Logistic | | NA |
|  | Probability | Coefficient (95%CI) | P-value | Coefficient (95%CI) | P-value | Coefficient (95%CI) | P-value | Probability |
| Shape (intercept) | NA | 0.010 (0.009;0.012) | <0.01 | -0.016 (-0.021;-0.010) | <0.01 | 1.17 (1.06;1.29) | <0.01 | NA |
| Rate / Scale | NA | 0.000 (0.000;0.001) | 0.02 | 0.004 (0.003;0.005) | <0.01 | 1,390 (397;4,850) | 0.48 | NA |
| Age |  |  |  |  |  |  |  |  |
| ≤ 54 | 0.028 (0.004;0.052) | Reference |  |  |  | -3.439 (-4.363;-2.515) |  | 0.000 |
| 55-59 | 0.028 (0.004;0.052) | 0.632 (-0,045;1,308) |  |  |  | -3.766 (-4.778;-2.754) |  | 0.001 |
| 60-64 | 0.032 (0.011;0.053) | 0.591 (-0.037;1,219) |  |  |  | -4.094 (-5.194;-2.994) |  | 0.001 |
| 65-69 | 0.032 (0.011;0.053) | 1.039 (0.453;1.624) |  |  |  | -4.421 (-5.609;-3.233) |  | 0.001 |
| 70-74 | 0.064 (0.040;0.087) | 1.701 (1.150;2.252) | <0.01 | NA | NA | -4.749 (-6.025;-3.473) | <0.01 | 0.002 |
| 75-79 | 0.073 (0.050;0.097) | 2.159 (1.617;2.702) |  |  |  | -5.076 (-6.440;-3.712) |  | 0.003 |
| 80-84 | 0.114 (0.081;0.147) | 2.823 (2.281;3.365) |  |  |  | -5.404 (-6.856;-3.952) |  | 0.004 |
| 85-89 | 0.156 (0.099;0.213) | 3.166 (2.604;3.729) |  |  |  | -5.731 (-7.271;-4.191) |  | 0.005 |
| 90-95 | 0.333 (0.178;0.488) | 3.315 (2623;4.007) |  |  |  | -6.059 (-7.687;-4.431) |  | 0.006 |
| Lymph nodes evaluated (≥10 vs <10) | NA | NA | NA | -0.519 (-0.762;-0.276) | <0.01 | NA | NA | NA |
| pT stage (pT4 vs pT3) | NA | NA | NA | 1.470 (1.057;1.881) | <0.01 | NA | NA | NA |
| Tumor sidedness (Left vs right) | NA | NA | NA | 0.505 (0.272;0.737) | <0.01 | NA | NA | NA |
| MMR subgroups |  |  |  |  |  |  |  |  |
| *dMMR* | NA | NA | NA | -0.942 | NA | NA | NA | NA |
| *pMMR* | NA | NA | NA | 0.108 | NA | NA | NA | NA |
| ctDNA subgroups |  |  |  |  |  |  |  |  |
| *ctDNA-positive* | NA | NA | NA | 2.209 | NA | NA | NA | NA |
| *ctDNA-negative* | NA | NA | NA | -0.264 | NA | NA | NA | NA |
| Treatment effect ACT | NA | NA | NA | -0.313 | <0.01 | NA | NA | NA |
|  | NA | NA | NA |  | 0.13 | NA | NA | NA |
| Abbreviations: 90DM = 90-day mortality; DIAG = diagnosis, REC = recurrence, DOC = death other causes; NA = not applicable; pMMR = proficient mismatch repair; dMMR = deficient mismatch repair; ctDNA = circulating tumor DNA; ACT = adjuvant chemotherapy. a Transition REC-DOC and REC-DCC were estimated in the same parametric survival model. Transition REC-DCC is calculated as the difference of transition REC-DOC + REC-DCC and transition REC-DOC. | | | | | | | | |

Appenix 2: CHEERS 2022 Checklist

| **Topic** | **No.** | **Item** | **Location where item is reported** |
| --- | --- | --- | --- |
| **Title** | | | |
|  | 1 | Identify the study as an economic evaluation and specify the interventions being compared. | Title |
| **Abstract** | | | |
|  | 2 | Provide a structured summary that highlights context, key methods, results, and alternative analyses. | Abstract |
| **Introduction** | | | |
| **Background and objectives** | 3 | Give the context for the study, the study question, and its practical relevance for decision making in policy or practice. | Introduction |
| **Methods** | | | |
| **Health economic analysis plan** | 4 | Indicate whether a health economic analysis plan was developed and where available. | Not reported |
| **Study population** | 5 | Describe characteristics of the study population (such as age range, demographics, socioeconomic, or clinical characteristics). | Material and Methods (subsections "the PATTERN- model" and "inclusion of ctDNA status") |
| **Setting and location** | 6 | Provide relevant contextual information that may influence findings. | Material and Methods (subsections "the PATTERN- model" and "Inclusion of ctDNA status") |
| **Comparators** | 7 | Describe the interventions or strategies being compared and why chosen. | Material and Methods (subsection "Strategies") |
| **Perspective** | 8 | State the perspective(s) adopted by the study and why chosen. | Material and Methods (subsection "Costs and health utilities") |
| **Time horizon** | 9 | State the time horizon for the study and why appropriate. | Material and Methods (subsection "the PATTERN- model") |
| **Discount rate** | 10 | Report the discount rate(s) and reason chosen. | Material and Methods (subsection "Costs and health utilities") |
| **Selection of outcomes** | 11 | Describe what outcomes were used as the measure(s) of benefit(s) and harm(s). | Material and Methods (subsections "Costs and health utilities" and "Outcomes") |

1

| **Topic** | **No.** | **Item** | **Location where item is reported** |
| --- | --- | --- | --- |
| **Measurement of outcomes** | 12 | Describe how outcomes used to capture benefit(s) and harm(s) were measured. | Material and Methods (subsections "Costs and health utilities" and "Outcomes") |
| **Valuation of outcomes** | 13 | Describe the population and methods used to measure and value outcomes. | Material and Methods (subsections "Costs and health utilities" and "Outcomes") |
| **Measurement and valuation of resources and costs** | 14 | Describe how costs were valued. | Material and Methods (subsection "Costs and health utilities") |
| **Currency, price date, and conversion** | 15 | Report the dates of the estimated resource quantities and unit costs, plus the currency and year of conversion. | Material and Methods (subsection "Costs and health utilities") |
| **Rationale and description of model** | 16 | If modelling is used, describe in detail and why used. Report if the model is publicly available and where it can be accessed. | Material and Methods (subsection "the PATTERN- model") |
| **Analytics and assumptions** | 17 | Describe any methods for analysing or statistically transforming data, any extrapolation methods, and approaches for validating any model used. | Material and Methods (subsection "the PATTERN- model") |
| **Characterising heterogeneity** | 18 | Describe any methods used for estimating how the results of the study vary for subgroups. | Material and Methods (subsections "the PATTERN- model" and "inclusion of ctDNA status") |
| **Characterising distributional effects** | 19 | Describe how impacts are distributed across different individuals or adjustments made to reflect priority populations. | Material and Methods (subsection "the PATTERN- model") |
| **Characterising uncertainty** | 20 | Describe methods to characterise any sources of uncertainty in the analysis. | Material and Methods (subsection "Sensitivity analyses") |
| **Approach to engagement with patients and others affected by the study** | 21 | Describe any approaches to engage patients or service recipients, the general public, communities, or stakeholders (such as clinicians or payers) in the design of the study. | Not reported |
| **Results** | | | |
| **Study parameters** | 22 | Report all analytic inputs (such as values, ranges, references) including uncertainty or distributional assumptions. | Material and Methods (subsections "the PATTERN- model" and "inclusion of ctDNA status") |
| **Summary of main results** | 23 | Report the mean values for the main categories of costs and outcomes of interest and summarise them in the most appropriate overall measure. | Results (subsection "Base-case analysis") |
| **Effect of uncertainty** | 24 | Describe how uncertainty about analytic judgments, inputs, or projections affect findings. Report the effect of choice of discount rate and time horizon, if applicable. | Results (subsection "Sensitivity analyses") |

2

| **Topic** | **No.** | **Item** | **Location where item is reported** |
| --- | --- | --- | --- |
| **Effect of engagement with patients and others affected by the study** | 25 | Report on any difference patient/service recipient, general public, community, or stakeholder involvement made to the approach or findings of the study | Not reported |
| **Discussion** | | | |
| **Study findings, limitations, generalisability, and current knowledge** | 26 | Report key findings, limitations, ethical or equity considerations not captured, and how these could affect patients, policy, or practice. | Discussion |
| **Other relevant information** | | | |
| **Source of funding** | 27 | Describe how the study was funded and any role of the funder in the identification, design, conduct, and reporting of the analysis | Conflict of Interest |
| **Conflicts of interest** | 28 | Report authors conflicts of interest according to journal or International Committee of Medical Journal Editors requirements. | Funding |

*From:* Husereau D, Drummond M, Augustovski F, et al. Consolidated Health Economic Evaluation Reporting Standards 2022 (CHEERS 2022) Explanation and Elaboration: A Report of the ISPOR CHEERS II Good Practices Task Force. Value Health 2022;25. doi:10.1016/j.jval.2021.10.008

3

# Supplementary Materials 3. Input parameters for sensitivity analyses

| **Sensitivity analysis** | **Adapted parameter(s)** | **Input base-case analysis** | **Input sensitivity analysis** |
| --- | --- | --- | --- |
| **Cost of ctDNA test** | Cost of ctDNA test | €2400 | €1000  €1250  €1500  €1750  €2000 |
| **Strategy adherence** | Proportion of high-risk group who receives ACT | 100% | Patients <75 years: 44%; ≥75 years: 11% |
| **Treatment effect ACT** | HR for treatment effect of ACT | -0.313 for all patients | ctDNA-positive: -0.632, ctDNA-negative: -0.253  ctDNA-positive: -1.104, ctDNA-negative: -0.174 |
| **Test performance** | Proportion of ctDNA-positive patients ; coefficient (transition DIAG-REC) for ctDNA-negative | 5.8% ; -0.264 | 8% ; -0.3895  10% ; -0.523  12% ; -0.685 |

ACT adjuvant chemotherapy; ctDNA circulating tumor DNA; HR hazard ratio

# Supplementary Materials 4. Results base-case analysis

1. Base-case analysis results cost-effectiveness analysis

| **Strategy** | **% received ACT** | **Recurrences** | **CC deaths** | **LY** | **QALY** | **Costs** | **NMB** | **incremental ICER** |
| --- | --- | --- | --- | --- | --- | --- | --- | --- |
| No ACT | 0.00% | 163 | 138 | 9.755 | 8.027 | € 26,227 | € 375,131 | ref |
| Current guideline  (pT4, pMMR) | 10.98% | 155 | 131 | 9.815 | 8.079 | € 26,911 | € 377,033 | € 13,223 |
| ctDNA-only  (ctDNA-positive) | 5.81% | 158 | 134 | 9.795 | 8.063 | € 28,735 | € 374,392 | Dominated |
| Combination 1 (pT4, pMMR and  ctDNA-positive,pMMR) | 15.24% | 151 | 127 | 9.849 | 8.108 | € 28,970 | € 376,440 | Dominated* |
| Combination 2 (pT4, pMMR and  ctDNA-positive) | 16.16% | 150 | 127 | 9.854 | 8.113 | € 29,402 | € 376,244 | Dominated* |
| Combination 3 (pT4 and  ctDNA-positive) | 18.10% | 149 | 126 | 9.859 | 8.117 | € 29,481 | € 376,370 | € 67,413 |

CC colon cancer; QALYs Quality-adjusted life years; ACT adjuvant chemotherapy; pMMR proficient mismatch repair; ctDNA circulating tumor DNA; NMB Net Monetary Benefit; ICER incremental cost-effectiveness ratio

# Distribution of recurrences over the risk groups

|  | **Overall** | | **High-risk group (received ACT)** | | | | **Low-risk group (untreated)** | | | |
| --- | --- | --- | --- | --- | --- | --- | --- | --- | --- | --- |
| **Strategy** | Total recurrences | Recurrence rate overall | Proportion high-risk | Recurrences in high-risk group | Recurrence rate in high-risk  group | % of all recurrences  that is high-risk | Proportion low- risk | Recurrences in low-risk group | Recurrence rate in low-risk  group | % of all recurrences  that is low-risk |
| No ACT | 163 | 16.3% |  | | | |  | | | |
| Current guideline  (pT4, pMMR) | 155 | 15.5% | 10.98% | 36 | 33.1% | 24% | 89.02% | 118 | 13.3% | 76% |
| ctDNA-only  (ctDNA-positive) | 158 | 15.8% | 5.81% | 34 | 59.1% | 22% | 94.19% | 124 | 13.2% | 78% |
| Combination 1 (pT4, pMMR and  ctDNA-positive,pMMR) | 151 | 15.1% | 15.24% | 62 | 40.6% | 41% | 84.76% | 89 | 10.5% | 59% |
| Combination 2 (pT4, pMMR and  ctDNA-positive) | 150 | 15.0% | 16.16% | 65 | 40.3% | 43% | 83.84% | 85 | 10.1% | 57% |
| Combination 3 (pT4 and  ctDNA-positive) | 149 | 14.9% | 18.10% | 67 | 37.2% | 45% | 81.90% | 82 | 10.0% | 55% |

ACT adjuvant chemotherapy; pMMR proficient mismatch repair; ctDNA circulating tumor DNA;

# Supplementary Materials 5. Sensitivity analysis: costs of ctDNA testing.

|  | **Effects** | | | | **Costs = 1000** | | | **Costs = 1250** | | | **Costs = 1500** | | | **Costs = 1750** | | | **Costs = 2000** | | |
| --- | --- | --- | --- | --- | --- | --- | --- | --- | --- | --- | --- | --- | --- | --- | --- | --- | --- | --- | --- |
| **Strategy** | **% received ACT** | **REC** | **CC deaths** | **QALYs** | **Costs** | **NMB** | **ICER** | **Costs** | **NMB** | **ICER** | **Costs** | **NMB** | **ICER** | **Costs** | **NMB** | **ICER** | **Costs** | **NMB** | **ICER** |
| No ACT | 0.00% | 163 | 138 | 8.027 | € 26,227 | € 375,131 | ref | € 26,227 | € 375,131 | ref | € 26,227 | € 375,131 | ref | € 26,227 | € 375,131 | ref | € 26,227 | € 375,131 | ref |
| Current guideline  (pT4, pMMR) | 10.98% | 155 | 131 | 8.079 | € 26,911 | € 377,033 | € 13,223 | € 26,911 | € 377,033 | € 13,223 | € 26,911 | € 377,033 | € 13,223 | € 26,911 | € 377,033 | € 13,223 | € 26,911 | € 377,033 | € 13,223 |
| ctDNA-only  (ctDNA-positive) | 5.81% | 158 | 134 | 8.063 | € 27,335 | € 375,792 | Dominated | € 27,585 | € 375,542 | Dominated | € 27,835 | € 375,292 | Dominated | € 28,085 | € 375,042 | Dominated | € 28,335 | € 374,792 | Dominated |
| Combination 1 (pT4, pMMR and ctDNA-  positive,pMMR) | 15.24% | 151 | 127 | 8.108 | € 27,791 | € 377,618 | € 30,030 | € 28,002 | € 377,408 | € 37,214 | € 28,212 | € 377,197 | Dominated* | € 28,423 | € 376,987 | Dominated* | € 28,633 | € 376,776 | Dominated* |
| Combination 2 (pT4, pMMR and  ctDNA-positive) | 16.16% | 150 | 127 | 8.113 | € 28,002 | € 377,644 | Dominated* | € 28,252 | € 377,394 | Dominated* | € 28,502 | € 377,144 | Dominated* | € 28,752 | € 376,894 | Dominated* | € 29,002 | € 376,644 | Dominated* |
| Combination 3 (pT4 and  ctDNA-positive) | 18.10% | 149 | 126 | 8.117 | € 28,081 | € 377,770 | € 32,844 | € 28,331 | € 377,520 | € 37,328 | € 28,581 | € 377,270 | € 43,799 | € 28,831 | € 377,020 | € 50,359 | € 29,081 | € 376,770 | € 56,918 |

QALYs Quality-adjusted life years; ACT adjuvant chemotherapy; pMMR proficient mismatch repair; ctDNA circulating tumor DNA; NMB Net Monetary Benefit; ICER incremental cost-effectiveness ratio

# Supplementary Materials 6. Sensitivity -analysis: strategy adherence (realistic adherence)

| **Strategy** | **% received ACT** | **Recurrences** | **CC deaths** | **LYs** | **QALY** | **Costs** | **NMB** | **incremental ICER** |
| --- | --- | --- | --- | --- | --- | --- | --- | --- |
| No ACT | 0.00% | 163 | 138 | 9.755 | 8.027 | € 26,227 | € 375,131 | ref |
| Current guideline  (pT4, pMMR) | 3.17% | 161 | 136 | 9.776 | 8.046 | € 26,530 | € 375,749 | € 16,438 |
| ctDNA-only  (ctDNA-positive) | 1.78% | 162 | 137 | 9.769 | 8.040 | € 28,664 | € 373,333 | Dominated |
| Combination 1 (pT4, pMMR and  ctDNA-positive,pMMR) | 4.49% | 159 | 135 | 9.788 | 8.056 | € 28,562 | € 374,247 | Dominated* |
| Combination 2 (pT4, pMMR and  ctDNA-positive) | 4.77% | 159 | 135 | 9.790 | 8.058 | € 28,961 | € 373,935 | Dominated* |
| Combination 3 (pT4 and  ctDNA-positive) | 5.33% | 159 | 134 | 9.792 | 8.059 | € 28,944 | € 374,027 | € 174,345 |

CC colon cancer; QALYs Quality-adjusted life years; ACT adjuvant chemotherapy; pMMR proficient mismatch repair; ctDNA circulating tumor DNA; NMB Net Monetary Benefit; ICER incremental cost-effectiveness ratio

# Supplementary Materials 7. Sensitivity -analysis: treatment effect ACT

1. Results cost-effectiveness analysis

| **Strategy** | **% received ACT** | **Recurrences** | **CC deaths** | **LY** | **QALY** | **Costs** | **NMB** | **incremental ICER** |
| --- | --- | --- | --- | --- | --- | --- | --- | --- |
| **HR of treatment effect**  **ctDNA-positive patients: 0.531** |  |  |  |  |  |  |  |  |
| No ACT | 0.00% | 163 | 138 | 9.755 | 8.027 | € 26,227 | € 375,131 | ref |
| Current guideline  (pT4, pMMR) | 10.98% | 156 | 132 | 9.807 | 8.071 | € 26,964 | € 376,609 | € 16,626 |
| ctDNA-only  (ctDNA-positive) | 5.81% | 153 | 129 | 9.837 | 8.100 | € 28,513 | € 376,497 | Dominated* |
| Combination 1 (pT4, pMMR and  ctDNA-positive,pMMR) | 15.24% | 148 | 125 | 9.875 | 8.132 | € 28,837 | € 377,752 | € 31,059 |
| Combination 2 (pT4, pMMR and  ctDNA-positive) | 16.16% | 146 | 124 | 9.885 | 8.141 | € 29,242 | € 377,807 | Dominated* |
| Combination 3 (pT4 and  ctDNA-positive) | 18.10% | 146 | 123 | 9.889 | 8.144 | € 29,325 | € 377,886 | € 39,222 |
| **HR of treatment effect**  **ctDNA-positive patients: 0.331** |  |  |  |  |  |  |  |  |
| No ACT | 0.00% | 163 | 138 | 9.755 | 8.027 | € 26,227 | € 375,131 | ref |
| Current guideline  (pT4, pMMR) | 10.98% | 158 | 133 | 9.798 | 8.063 | € 27,024 | € 376,120 | Dominated* |
| ctDNA-only  (ctDNA-positive) | 5.81% | 146 | 123 | 9.892 | 8.151 | € 28,205 | € 379,354 | € 15,945 |
| Combination 1 (pT4, pMMR and  ctDNA-positive,pMMR) | 15.24% | 143 | 121 | 9.910 | 8.164 | € 28,647 | € 379,560 | Dominated* |
| Combination 2 (pT4, pMMR and  ctDNA-positive) | 16.16% | 141 | 119 | 9.926 | 8.179 | € 29,019 | € 379,912 | € 29,674 |
| Combination 3 (pT4 and  ctDNA-positive) | 18.10% | 141 | 119 | 9.929 | 8.181 | € 29,110 | € 379,925 | € 43,923 |

CC colon cancer; QALYs Quality-adjusted life years; ACT adjuvant chemotherapy; pMMR proficient mismatch repair; ctDNA circulating tumor DNA; HR hazard ratio; NMB Net Monetary Benefit; ICER incremental cost- effectiveness ratio

# Distribution of recurrences over the risk groups

|  | **Overall** | | **High-risk group**  **(received ACT)** | | | | **Low-risk group**  **(untreated)** | | | |
| --- | --- | --- | --- | --- | --- | --- | --- | --- | --- | --- |
| **Strategy** | Total recurrences | Recurrence rate overall | Proportion high- risk | Recurrences in high-risk group | Recurrence rate in high-risk group | % of all recurrences that  is high-risk | Proportion low- risk | Recurrences in low-risk group | Recurrence rate in low-risk group | % of all recurrences that  is low-risk |
| **HR of treatment effect**  **ctDNA-positive patients: 0.531** |  | |  | | | |  | | | |
| No ACT | 163 | 16.32% |  | | | |  | | | |
| Current guideline  (pT4, pMMR) | 156 | 15.60% | 10.98% | 38 | 34.3% | 24% | 89.02% | 118 | 13.3% | 76% |
| ctDNA-only  (ctDNA-positive) | 153 | 15.31% | 5.81% | 29 | 50.2% | 19% | 94.19% | 124 | 13.2% | 81% |
| Combination 1 (pT4, pMMR and  ctDNA-positive,pMMR) | 148 | 14.77% | 15.24% | 59 | 38.6% | 40% | 84.76% | 89 | 10.5% | 60% |
| Combination 2 (pT4, pMMR and  ctDNA-positive) | 146 | 14.63% | 16.16% | 61 | 38.0% | 42% | 83.84% | 85 | 10.1% | 58% |
| Combination 3 (pT4 and  ctDNA-positive) | 146 | 14.56% | 18.10% | 64 | 35.2% | 44% | 81.90% | 82 | 10.0% | 56% |
| **HR of treatment effect**  **ctDNA-positive patients: 0.331** |  | |  | | | |  | | | |
| No ACT | 163 | 16.32% |  | | | |  | | | |
| Current guideline  (pT4, pMMR) | 158 | 15.75% | 10.98% | 39 | 35.7% | 25% | 89.02% | 118 | 13.3% | 75% |
| ctDNA-only  (ctDNA-positive) | 146 | 14.58% | 5.81% | 22 | 37.8% | 15% | 94.19% | 124 | 13.2% | 85% |
| Combination 1 (pT4, pMMR and  ctDNA-positive,pMMR) | 143 | 14.33% | 15.24% | 54 | 35.7% | 38% | 84.76% | 89 | 10.5% | 62% |
| Combination 2 (pT4, pMMR and  ctDNA-positive) | 141 | 14.11% | 16.16% | 56 | 34.8% | 40% | 83.84% | 85 | 10.1% | 60% |
| Combination 3 (pT4 and  ctDNA-positive) | 141 | 14.06% | 18.10% | 59 | 32.5% | 42% | 81.90% | 82 | 10.0% | 58% |

ACT adjuvant chemotherapy; pMMR proficient mismatch repair; ctDNA circulating tumor DNA

# Supplementary Materials 8. Sensitivity -analysis: performance ctDNA test

- 1. Results cost-effectiveness analysis

| **Strategy** | **% received ACT** | **Recurrences** | **CC deaths** | **LY** | **QALY** | **Costs** | **NMB** | **incremental ICER** |
| --- | --- | --- | --- | --- | --- | --- | --- | --- |
| **Test performance 8%** |  |  |  |  |  |  |  |  |
| No ACT | 0.00% | 163 | 138 | 9.755 | 8.027 | € 26,227 | € 375,131 | ref |
| Current guideline  (pT4, pMMR) | 10.98% | 155 | 131 | 9.815 | 8.079 | € 26,911 | € 377,033 | € 13,223 |
| ctDNA-only  (ctDNA-positive) | 8.00% | 156 | 132 | 9.809 | 8.074 | € 28,765 | € 374,957 | Dominated |
| Combination 1 (pT4, pMMR and  ctDNA-positive,pMMR) | 16.84% | 149 | 126 | 9.854 | 8.113 | € 28,993 | € 376,679 | Dominated* |
| Combination 2 (pT4, pMMR and  ctDNA-positive) | 18.11% | 148 | 125 | 9.862 | 8.120 | € 29,445 | € 376,553 | Dominated* |
| Combination 3 (pT4 and  ctDNA-positive) | 20.00% | 147 | 125 | 9.867 | 8.123 | € 29,524 | € 376,650 | € 58,593 |
| **Test performance 10%** |  |  |  |  |  |  |  |  |
| No ACT | 0.00% | 163 | 138 | 9.755 | 8.027 | € 26,227 | € 375,131 | ref |
| Current guideline  (pT4, pMMR) | 10.98% | 155 | 131 | 9.815 | 8.079 | € 26,911 | € 377,033 | € 13,223 |
| ctDNA-only  (ctDNA-positive) | 10.00% | 153 | 130 | 9.821 | 8.086 | € 28,790 | € 375,488 | Dominated* |
| Combination 1 (pT4, pMMR and  ctDNA-positive,pMMR) | 18.31% | 148 | 125 | 9.859 | 8.118 | € 29,015 | € 376,889 | Dominated* |
| Combination 2 (pT4, pMMR and  ctDNA-positive) | 19.89% | 146 | 124 | 9.869 | 8.126 | € 29,485 | € 376,827 | Dominated* |
| Combination 3 (pT4 and  ctDNA-positive) | 21.74% | 146 | 123 | 9.873 | 8.129 | € 29,564 | € 376,897 | € 52,702 |
| **Test performance 12%** |  |  |  |  |  |  |  |  |
| No ACT | 0.00% | 163 | 138 | 9.755 | 8.027 | € 26,227 | € 375,131 | ref |
| Current guideline  (pT4, pMMR) | 10.98% | 155 | 131 | 9.815 | 8.079 | € 26,911 | € 377,033 | € 13,223 |
| ctDNA-only  (ctDNA-positive) | 12.00% | 151 | 127 | 9.836 | 8.099 | € 28,800 | € 376,151 | Dominated* |
| Combination 1 (pT4, pMMR and  ctDNA-positive,pMMR) | 19.77% | 146 | 124 | 9.866 | 8.125 | € 29,025 | € 377,201 | Dominated* |
| Combination 2 (pT4, pMMR and  ctDNA-positive) | 21.67% | 145 | 122 | 9.878 | 8.134 | € 29,513 | € 377,202 | Dominated* |

| Combination 3 (pT4 and  ctDNA-positive) | 23.48% | 144 | 122 | 9.881 | 8.137 | € 29,591 | € 377,245 | € 46,342 |
| --- | --- | --- | --- | --- | --- | --- | --- | --- |

CC colon cancer; QALYs Quality-adjusted life years; ACT adjuvant chemotherapy; pMMR proficient mismatch repair; ctDNA circulating tumor DNA; NMB Net Monetary Benefit; ICER incremental cost-effectiveness ratio

# Distribution of recurrences over the risk groups

|  | **Overall** | | **High-risk group (received ACT)** | | | | **Low-risk group (untreated)** | | | |
| --- | --- | --- | --- | --- | --- | --- | --- | --- | --- | --- |
| **Strategy** | Total recurrences | Recurrence rate overall | Proportion high- risk | Recurrences in high-risk group | Recurrence rate in high-risk group | % of all recurrences that is high-risk | Proportion low- risk | Recurrences in low-risk group | Recurrence rate in low-risk group | % of all recurrences that is low-risk |
| **Test performance 8%** |  | |  | | | |  | | | |
| No ACT | 163 | 16.3% |  | | | |  | | | |
| Current guideline  (pT4, pMMR) | 155 | 15.5% | 10.98% | 36 | 33.1% | 24% | 89.02% | 118 | 13.3% | 76% |
| ctDNA-only  (ctDNA-positive) | 156 | 15.6% | 8.00% | 47 | 59.1% | 30% | 92.00% | 108 | 11.8% | 70% |
| Combination 1 (pT4, pMMR and  ctDNA-positive,pMMR) | 149 | 14.9% | 16.84% | 70 | 41.6% | 47% | 83.16% | 79 | 9.5% | 53% |
| Combination 2 (pT4, pMMR and  ctDNA-positive) | 148 | 14.8% | 18.11% | 74 | 41.1% | 50% | 81.89% | 74 | 9.0% | 50% |
| Combination 3 (pT4 and  ctDNA-positive) | 147 | 14.7% | 20.00% | 76 | 38.2% | 52% | 80.00% | 71 | 8.9% | 48% |
| **Test performance 10%** |  | |  | | | |  | | | |
| No ACT | 163 | 16.3% |  | | | |  | | | |
| Current guideline  (pT4, pMMR) | 155 | 15.5% | 10.98% | 36 | 33.1% | 24% | 89.02% | 118 | 13.3% | 76% |
| ctDNA-only  (ctDNA-positive) | 153 | 15.3% | 10.00% | 59 | 59.1% | 39% | 90.00% | 94 | 10.5% | 61% |
| Combination 1 (pT4, pMMR and  ctDNA-positive,pMMR) | 148 | 14.8% | 18.31% | 77 | 42.3% | 52% | 81.69% | 70 | 8.6% | 48% |
| Combination 2 (pT4, pMMR and  ctDNA-positive) | 146 | 14.6% | 19.89% | 83 | 41.7% | 57% | 80.11% | 64 | 7.9% | 43% |
| Combination 3 (pT4 and  ctDNA-positive) | 146 | 14.6% | 21.74% | 85 | 38.9% | 58% | 78.26% | 61 | 7.8% | 42% |
| **Test performance 12%** |  | |  | | | |  | | | |
| No ACT | 163 | 16.3% |  | | | |  | | | |

| Current guideline  (pT4, pMMR) | 155 | 15.5% | 10.98% | 36 | 33.1% | 24% | 89.02% | 118 | 13.3% | 76% |
| --- | --- | --- | --- | --- | --- | --- | --- | --- | --- | --- |
| ctDNA-only  (ctDNA-positive) | 151 | 15.1% | 12.00% | 151 | 59.1% | 47% | 88.00% | 80 | 9.0% | 53% |
| Combination 1 (pT4, pMMR and  ctDNA-positive,pMMR) | 146 | 14.6% | 19.77% | 146 | 42.8% | 58% | 80.23% | 61 | 7.7% | 42% |
| Combination 2 (pT4, pMMR and  ctDNA-positive) | 145 | 14.5% | 21.67% | 145 | 42.1% | 63% | 78.33% | 53 | 6.8% | 37% |
| Combination 3 (pT4 and  ctDNA-positive) | 144 | 14.4% | 23.48% | 144 | 39.5% | 64% | 76.52% | 51 | 6.7% | 36% |

ACT adjuvant chemotherapy; pMMR proficient mismatch repair; ctDNA circulating tumor DNA

# Supplementary Materials 9. Distribution of the recurrences over the risk-groups in the absence of ACT.

- - 1.
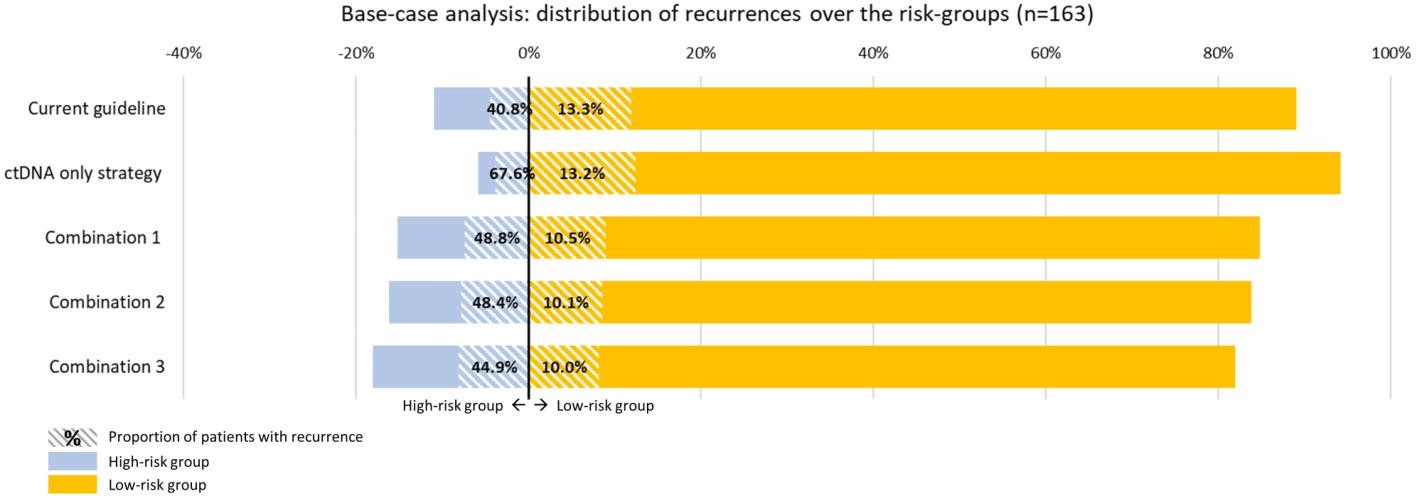
Base-case analysis

Combination 1: pT4, pMMR patients and ctDNA-positive,pMMR patients. Combination 2: pT4, pMMR patients and ctDNA-positive patients. Combination 3: pT4 patients and ctDNA-positive patients.

#
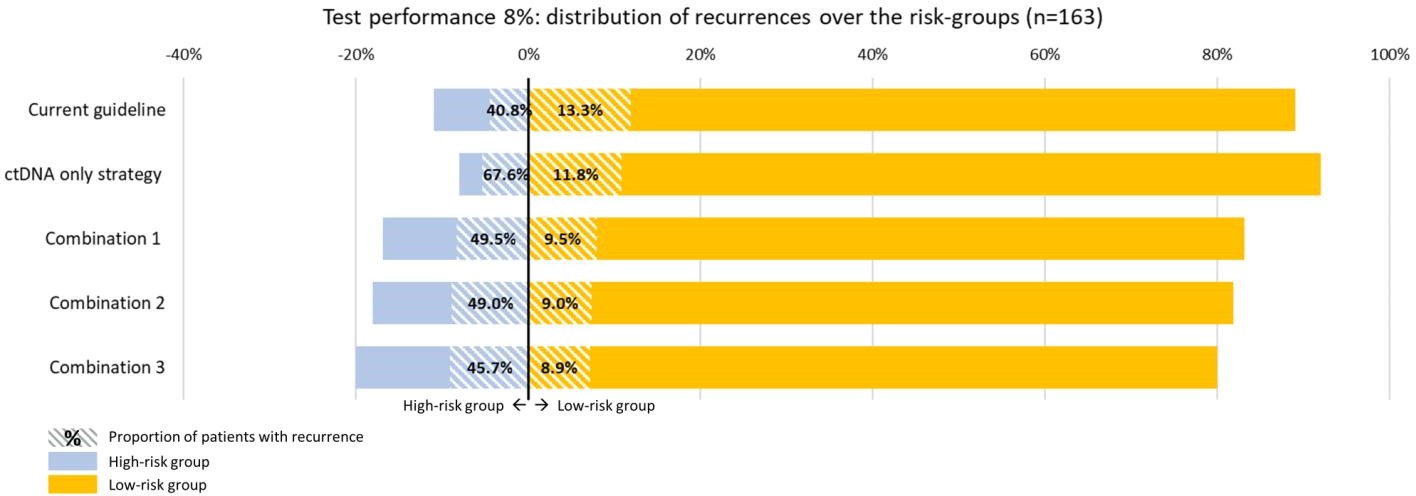
Sensitivity analysis: Test performance 8%

Combination 1: pT4, pMMR patients and ctDNA-positive,pMMR patients. Combination 2: pT4, pMMR patients and ctDNA-positive patients. Combination 3: pT4 patients and ctDNA-positive patients.

#
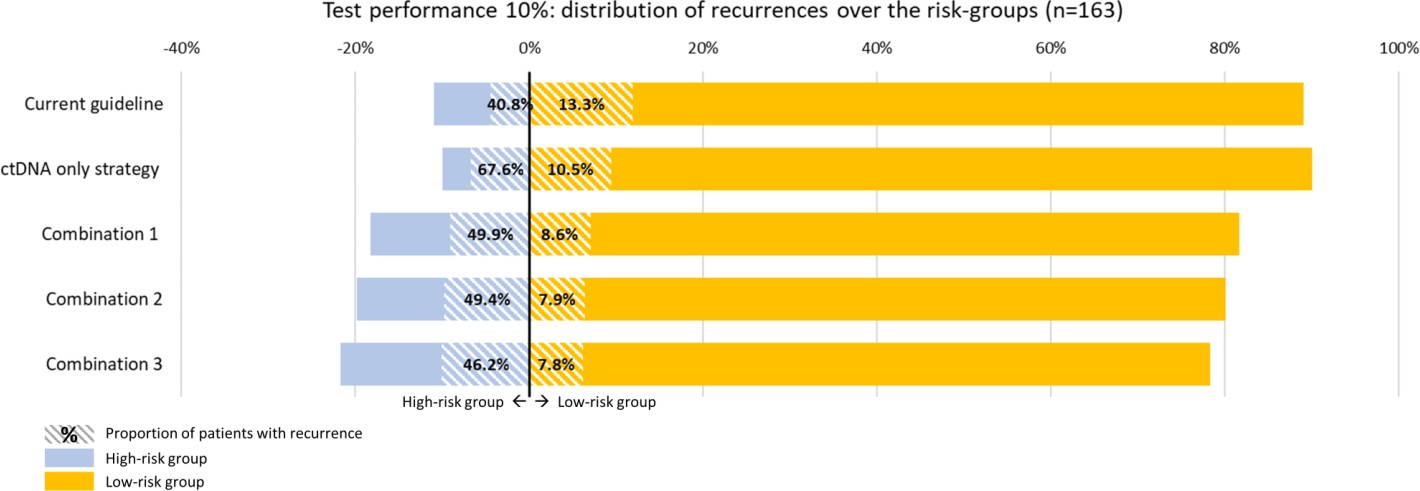
Sensitivity analysis: Test performance 10%

Combination 1: pT4, pMMR patients and ctDNA-positive,pMMR patients. Combination 2: pT4, pMMR patients and ctDNA-positive patients. Combination 3: pT4 patients and ctDNA-positive patients.

#
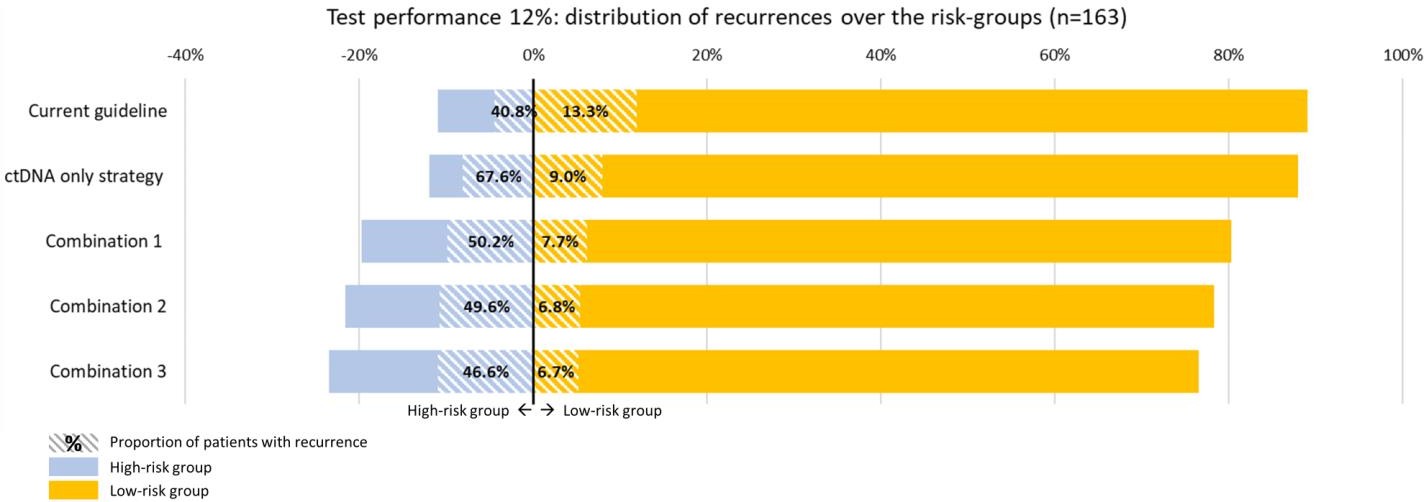
Sensitivity analysis: Test performance 12%

Combination 1: pT4, pMMR patients and ctDNA-positive,pMMR patients. Combination 2: pT4, pMMR patients and ctDNA-positive patients. Combination 3: pT4 patients and ctDNA-positive patients.

# Supplementary Materials 10. Transition matrices for base-case analysis.

90D-Death: 90-day mortality, DOC: Death due to other causes, DCC: Death due to colon cancer

| **Strategy: No ACT** | | | | | |
| --- | --- | --- | --- | --- | --- |
| **Year** | **Diagnosis** | **90D-Death** | **Recurrence** | **DOC** | **DCC** |
| 1 | 1000.0 | 0.0 | 0.0 | 0.0 | 0.0 |
| 2 | 859.7 | 67.3 | 34.9 | 23.6 | 14.6 |
| 3 | 796.4 | 67.3 | 43.0 | 55.7 | 37.6 |
| 4 | 741.8 | 67.3 | 43.8 | 88.5 | 58.5 |
| 5 | 692.7 | 67.3 | 41.8 | 122.2 | 76.1 |
| 6 | 647.3 | 67.3 | 38.7 | 156.2 | 90.5 |
| 7 | 604.5 | 67.3 | 35.4 | 190.6 | 102.3 |
| 8 | 563.7 | 67.3 | 32.1 | 225.1 | 111.8 |
| 9 | 522.9 | 67.3 | 29.0 | 261.4 | 119.4 |
| 10 | 485.8 | 67.3 | 26.1 | 295.4 | 125.4 |
| 11 | 450.0 | 67.3 | 23.6 | 328.9 | 130.2 |
| 12 | 417.6 | 67.3 | 19.5 | 362.1 | 133.5 |
| 13 | 385.8 | 67.3 | 16.8 | 394.8 | 135.4 |
| 14 | 352.8 | 67.3 | 14.6 | 428.8 | 136.4 |
| 15 | 323.4 | 67.3 | 13.0 | 459.2 | 137.1 |
| 16 | 294.6 | 67.3 | 11.8 | 488.9 | 137.5 |
| 17 | 266.7 | 67.3 | 10.8 | 517.6 | 137.7 |
| 18 | 239.8 | 67.3 | 9.9 | 545.2 | 137.9 |
| 19 | 213.3 | 67.3 | 8.8 | 572.6 | 137.9 |
| 20 | 189.3 | 67.3 | 8.2 | 597.3 | 138.0 |
| 21 | 166.5 | 67.3 | 7.6 | 620.6 | 138.0 |
| 22 | 145.0 | 67.3 | 7.2 | 642.5 | 138.0 |
| 23 | 125.0 | 67.3 | 6.9 | 662.9 | 138.0 |
| 24 | 105.7 | 67.3 | 6.0 | 683.0 | 138.0 |
| 25 | 89.3 | 67.3 | 5.7 | 699.6 | 138.0 |
| 26 | 74.4 | 67.3 | 5.5 | 714.9 | 138.0 |
| 27 | 60.9 | 67.3 | 5.3 | 728.5 | 138.0 |
| 28 | 49.1 | 67.3 | 5.0 | 740.6 | 138.0 |
| 29 | 38.5 | 67.3 | 4.1 | 752.2 | 138.0 |
| 30 | 29.9 | 67.3 | 4.0 | 760.9 | 138.0 |
| 31 | 22.7 | 67.3 | 3.8 | 768.2 | 138.0 |
| 32 | 16.8 | 67.3 | 3.7 | 774.3 | 138.0 |
| 33 | 12.1 | 67.3 | 3.5 | 779.1 | 138.0 |
| 34 | 8.2 | 67.3 | 2.6 | 784.0 | 138.0 |
| 35 | 5.7 | 67.3 | 2.5 | 786.6 | 138.0 |
| 36 | 3.8 | 67.3 | 2.4 | 788.5 | 138.0 |
| 37 | 2.4 | 67.3 | 2.3 | 790.0 | 138.0 |
| 38 | 1.5 | 67.3 | 2.3 | 791.0 | 138.0 |
| 39 | 0.8 | 67.3 | 1.5 | 792.4 | 138.0 |
| 40 | 0.5 | 67.3 | 1.4 | 792.8 | 138.0 |

| **Strategy: Current guideline** | | | | | |
| --- | --- | --- | --- | --- | --- |
| **Year** | **Diagnosis** | **90D-Death** | **Recurrence** | **DOC** | **DCC** |
| 1 | 1000,0 | 0,0 | 0,0 | 0,0 | 0,0 |
| 2 | 863,2 | 67,3 | 32,5 | 23,6 | 13,4 |
| 3 | 801,4 | 67,3 | 40,5 | 55,8 | 35,0 |
| 4 | 747,6 | 67,3 | 41,4 | 88,9 | 54,8 |
| 5 | 698,8 | 67,3 | 39,6 | 122,7 | 71,5 |
| 6 | 653,6 | 67,3 | 36,8 | 157,0 | 85,2 |
| 7 | 610,7 | 67,3 | 33,7 | 191,7 | 96,5 |
| 8 | 569,9 | 67,3 | 30,6 | 226,6 | 105,6 |
| 9 | 528,8 | 67,3 | 27,7 | 263,3 | 112,9 |
| 10 | 491,4 | 67,3 | 25,0 | 297,6 | 118,7 |
| 11 | 455,2 | 67,3 | 22,6 | 331,5 | 123,4 |
| 12 | 422,5 | 67,3 | 18,6 | 365,0 | 126,5 |
| 13 | 390,3 | 67,3 | 16,0 | 398,1 | 128,3 |
| 14 | 356,9 | 67,3 | 13,9 | 432,5 | 129,3 |
| 15 | 327,1 | 67,3 | 12,4 | 463,2 | 130,0 |
| 16 | 298,0 | 67,3 | 11,2 | 493,1 | 130,3 |
| 17 | 269,7 | 67,3 | 10,3 | 522,1 | 130,6 |
| 18 | 242,5 | 67,3 | 9,4 | 550,0 | 130,7 |
| 19 | 215,8 | 67,3 | 8,4 | 577,7 | 130,8 |
| 20 | 191,5 | 67,3 | 7,8 | 602,6 | 130,8 |
| 21 | 168,4 | 67,3 | 7,3 | 626,2 | 130,8 |
| 22 | 146,7 | 67,3 | 6,9 | 648,3 | 130,8 |
| 23 | 126,4 | 67,3 | 6,5 | 668,9 | 130,8 |
| 24 | 107,0 | 67,3 | 5,7 | 689,1 | 130,8 |
| 25 | 90,4 | 67,3 | 5,5 | 706,0 | 130,8 |
| 26 | 75,2 | 67,3 | 5,2 | 721,4 | 130,8 |
| 27 | 61,7 | 67,3 | 5,0 | 735,2 | 130,8 |
| 28 | 49,6 | 67,3 | 4,8 | 747,4 | 130,8 |
| 29 | 38,9 | 67,3 | 3,9 | 759,0 | 130,8 |
| 30 | 30,2 | 67,3 | 3,8 | 767,8 | 130,8 |
| 31 | 23,0 | 67,3 | 3,6 | 775,2 | 130,8 |
| 32 | 17,0 | 67,3 | 3,5 | 781,3 | 130,8 |
| 33 | 12,3 | 67,3 | 3,4 | 786,2 | 130,8 |
| 34 | 8,3 | 67,3 | 2,4 | 791,1 | 130,8 |
| 35 | 5,8 | 67,3 | 2,4 | 793,7 | 130,8 |
| 36 | 3,9 | 67,3 | 2,3 | 795,7 | 130,8 |
| 37 | 2,5 | 67,3 | 2,2 | 797,1 | 130,8 |
| 38 | 1,5 | 67,3 | 2,1 | 798,2 | 130,8 |
| 39 | 0,8 | 67,3 | 1,4 | 799,6 | 130,8 |
| 40 | 0,5 | 67,3 | 1,3 | 800,0 | 130,8 |

| **Strategy: ctDNA-only** | | | | | |
| --- | --- | --- | --- | --- | --- |
| **Year** | **Diagnosis** | **90D-Death** | **Recurrence** | **DOC** | **DCC** |
| 1 | 1000,0 | 0,0 | 0,0 | 0,0 | 0,0 |
| 2 | 863,3 | 67,3 | 32,4 | 23,6 | 13,3 |
| 3 | 800,9 | 67,3 | 40,9 | 55,8 | 35,1 |
| 4 | 746,5 | 67,3 | 42,1 | 88,8 | 55,3 |
| 5 | 697,2 | 67,3 | 40,5 | 122,6 | 72,4 |
| 6 | 651,5 | 67,3 | 37,7 | 156,8 | 86,5 |
| 7 | 608,5 | 67,3 | 34,6 | 191,4 | 98,1 |
| 8 | 567,5 | 67,3 | 31,5 | 226,1 | 107,6 |
| 9 | 526,4 | 67,3 | 28,5 | 262,7 | 115,1 |
| 10 | 489,0 | 67,3 | 25,7 | 296,8 | 121,1 |
| 11 | 452,9 | 67,3 | 23,2 | 330,6 | 126,0 |
| 12 | 420,4 | 67,3 | 19,2 | 363,9 | 129,2 |
| 13 | 388,3 | 67,3 | 16,5 | 396,8 | 131,1 |
| 14 | 355,2 | 67,3 | 14,3 | 431,0 | 132,2 |
| 15 | 325,5 | 67,3 | 12,8 | 461,6 | 132,8 |
| 16 | 296,5 | 67,3 | 11,5 | 491,4 | 133,2 |
| 17 | 268,4 | 67,3 | 10,5 | 520,3 | 133,5 |
| 18 | 241,4 | 67,3 | 9,7 | 548,0 | 133,6 |
| 19 | 214,7 | 67,3 | 8,6 | 575,6 | 133,7 |
| 20 | 190,5 | 67,3 | 8,0 | 600,4 | 133,7 |
| 21 | 167,6 | 67,3 | 7,5 | 623,9 | 133,7 |
| 22 | 145,9 | 67,3 | 7,1 | 645,9 | 133,7 |
| 23 | 125,8 | 67,3 | 6,7 | 666,4 | 133,7 |
| 24 | 106,4 | 67,3 | 5,9 | 686,6 | 133,7 |
| 25 | 89,9 | 67,3 | 5,6 | 703,4 | 133,7 |
| 26 | 74,9 | 67,3 | 5,4 | 718,7 | 133,7 |
| 27 | 61,3 | 67,3 | 5,1 | 732,5 | 133,7 |
| 28 | 49,4 | 67,3 | 4,9 | 744,6 | 133,7 |
| 29 | 38,7 | 67,3 | 4,0 | 756,2 | 133,7 |
| 30 | 30,1 | 67,3 | 3,9 | 765,0 | 133,7 |
| 31 | 22,8 | 67,3 | 3,7 | 772,4 | 133,7 |
| 32 | 16,9 | 67,3 | 3,6 | 778,4 | 133,7 |
| 33 | 12,2 | 67,3 | 3,5 | 783,3 | 133,7 |
| 34 | 8,3 | 67,3 | 2,5 | 788,2 | 133,7 |
| 35 | 5,7 | 67,3 | 2,4 | 790,8 | 133,7 |
| 36 | 3,8 | 67,3 | 2,3 | 792,8 | 133,7 |
| 37 | 2,5 | 67,3 | 2,3 | 794,2 | 133,7 |
| 38 | 1,5 | 67,3 | 2,2 | 795,2 | 133,7 |
| 39 | 0,8 | 67,3 | 1,4 | 796,7 | 133,7 |
| 40 | 0,5 | 67,3 | 1,4 | 797,1 | 133,7 |

| **Strategy: Combination 1** (pT4, pMMR and ctDNA-positive,pMMR) | | | | | |
| --- | --- | --- | --- | --- | --- |
| **Year** | **Diagnosis** | **90D-Death** | **Recurrence** | **DOC** | **DCC** |
| 1 | 1000,0 | 0,0 | 0,0 | 0,0 | 0,0 |
| 2 | 865,9 | 67,4 | 30,6 | 23,6 | 12,5 |
| 3 | 805,0 | 67,4 | 38,7 | 55,9 | 33,1 |
| 4 | 751,4 | 67,4 | 40,0 | 89,1 | 52,2 |
| 5 | 702,6 | 67,4 | 38,5 | 123,1 | 68,4 |
| 6 | 657,2 | 67,4 | 36,0 | 157,5 | 81,9 |
| 7 | 614,1 | 67,4 | 33,1 | 192,4 | 93,1 |
| 8 | 573,0 | 67,4 | 30,1 | 227,4 | 102,1 |
| 9 | 531,7 | 67,4 | 27,2 | 264,3 | 109,4 |
| 10 | 494,1 | 67,4 | 24,6 | 298,8 | 115,1 |
| 11 | 457,7 | 67,4 | 22,3 | 332,9 | 119,8 |
| 12 | 424,8 | 67,4 | 18,4 | 366,5 | 123,0 |
| 13 | 392,4 | 67,4 | 15,8 | 399,7 | 124,8 |
| 14 | 358,8 | 67,4 | 13,7 | 434,3 | 125,8 |
| 15 | 328,9 | 67,4 | 12,2 | 465,1 | 126,4 |
| 16 | 299,6 | 67,4 | 11,0 | 495,2 | 126,8 |
| 17 | 271,2 | 67,4 | 10,1 | 524,3 | 127,1 |
| 18 | 243,9 | 67,4 | 9,2 | 552,3 | 127,2 |
| 19 | 216,9 | 67,4 | 8,2 | 580,2 | 127,3 |
| 20 | 192,5 | 67,4 | 7,6 | 605,2 | 127,3 |
| 21 | 169,3 | 67,4 | 7,1 | 628,9 | 127,3 |
| 22 | 147,4 | 67,4 | 6,8 | 651,1 | 127,3 |
| 23 | 127,1 | 67,4 | 6,4 | 671,8 | 127,3 |
| 24 | 107,5 | 67,4 | 5,6 | 692,2 | 127,3 |
| 25 | 90,9 | 67,4 | 5,4 | 709,1 | 127,3 |
| 26 | 75,6 | 67,4 | 5,1 | 724,5 | 127,3 |
| 27 | 62,0 | 67,4 | 4,9 | 738,4 | 127,3 |
| 28 | 49,9 | 67,4 | 4,7 | 750,7 | 127,3 |
| 29 | 39,1 | 67,4 | 3,8 | 762,3 | 127,3 |
| 30 | 30,4 | 67,4 | 3,7 | 771,2 | 127,3 |
| 31 | 23,1 | 67,4 | 3,6 | 778,7 | 127,3 |
| 32 | 17,1 | 67,4 | 3,4 | 784,8 | 127,3 |
| 33 | 12,3 | 67,4 | 3,3 | 789,7 | 127,3 |
| 34 | 8,4 | 67,4 | 2,4 | 794,6 | 127,3 |
| 35 | 5,8 | 67,4 | 2,3 | 797,2 | 127,3 |
| 36 | 3,9 | 67,4 | 2,2 | 799,2 | 127,3 |
| 37 | 2,5 | 67,4 | 2,2 | 800,7 | 127,3 |
| 38 | 1,5 | 67,4 | 2,1 | 801,7 | 127,3 |
| 39 | 0,8 | 67,4 | 1,4 | 803,1 | 127,3 |
| 40 | 0,5 | 67,4 | 1,3 | 803,5 | 127,3 |

| **Strategy: Combination 2** (pT4, pMMR and ctDNA-positive) | | | | | |
| --- | --- | --- | --- | --- | --- |
| **Year** | **Diagnosis** | **90D-Death** | **Recurrence** | **DOC** | **DCC** |
| 1 | 1000,0 | 0,0 | 0,0 | 0,0 | 0,0 |
| 2 | 866,2 | 67,4 | 30,4 | 23,6 | 12,5 |
| 3 | 805,4 | 67,4 | 38,5 | 55,9 | 32,8 |
| 4 | 752,0 | 67,4 | 39,7 | 89,1 | 51,9 |
| 5 | 703,2 | 67,4 | 38,3 | 123,1 | 68,0 |
| 6 | 657,7 | 67,4 | 35,8 | 157,6 | 81,5 |
| 7 | 614,7 | 67,4 | 32,9 | 192,5 | 92,5 |
| 8 | 573,6 | 67,4 | 30,0 | 227,5 | 101,6 |
| 9 | 532,2 | 67,4 | 27,1 | 264,5 | 108,8 |
| 10 | 494,6 | 67,4 | 24,5 | 299,0 | 114,6 |
| 11 | 458,2 | 67,4 | 22,2 | 333,1 | 119,2 |
| 12 | 425,2 | 67,4 | 18,3 | 366,8 | 122,4 |
| 13 | 392,8 | 67,4 | 15,7 | 400,0 | 124,2 |
| 14 | 359,2 | 67,4 | 13,6 | 434,6 | 125,2 |
| 15 | 329,2 | 67,4 | 12,2 | 465,5 | 125,8 |
| 16 | 299,9 | 67,4 | 11,0 | 495,6 | 126,2 |
| 17 | 271,5 | 67,4 | 10,0 | 524,7 | 126,4 |
| 18 | 244,1 | 67,4 | 9,2 | 552,8 | 126,6 |
| 19 | 217,2 | 67,4 | 8,2 | 580,6 | 126,7 |
| 20 | 192,7 | 67,4 | 7,6 | 605,6 | 126,7 |
| 21 | 169,5 | 67,4 | 7,1 | 629,4 | 126,7 |
| 22 | 147,6 | 67,4 | 6,7 | 651,6 | 126,7 |
| 23 | 127,2 | 67,4 | 6,4 | 672,3 | 126,7 |
| 24 | 107,6 | 67,4 | 5,6 | 692,7 | 126,7 |
| 25 | 91,0 | 67,4 | 5,4 | 709,6 | 126,7 |
| 26 | 75,7 | 67,4 | 5,1 | 725,1 | 126,7 |
| 27 | 62,0 | 67,4 | 4,9 | 739,0 | 126,7 |
| 28 | 50,0 | 67,4 | 4,7 | 751,3 | 126,7 |
| 29 | 39,2 | 67,4 | 3,8 | 762,9 | 126,7 |
| 30 | 30,4 | 67,4 | 3,7 | 771,8 | 126,7 |
| 31 | 23,1 | 67,4 | 3,5 | 779,3 | 126,7 |
| 32 | 17,1 | 67,4 | 3,4 | 785,4 | 126,7 |
| 33 | 12,4 | 67,4 | 3,3 | 790,3 | 126,7 |
| 34 | 8,4 | 67,4 | 2,4 | 795,2 | 126,7 |
| 35 | 5,8 | 67,4 | 2,3 | 797,8 | 126,7 |
| 36 | 3,9 | 67,4 | 2,2 | 799,8 | 126,7 |
| 37 | 2,5 | 67,4 | 2,2 | 801,3 | 126,7 |
| 38 | 1,5 | 67,4 | 2,1 | 802,3 | 126,7 |
| 39 | 0,9 | 67,4 | 1,4 | 803,7 | 126,7 |
| 40 | 0,5 | 67,4 | 1,3 | 804,1 | 126,7 |

| **Strategy: Combination 3** (pT4 and ctDNA-positive) | | | | | |
| --- | --- | --- | --- | --- | --- |
| **Year** | **Diagnosis** | **90D-Death** | **Recurrence** | **DOC** | **DCC** |
| 1 | 1000,0 | 0,0 | 0,0 | 0,0 | 0,0 |
| 2 | 866,4 | 67,4 | 30,2 | 23,6 | 12,4 |
| 3 | 805,8 | 67,4 | 38,3 | 55,9 | 32,7 |
| 4 | 752,4 | 67,4 | 39,5 | 89,1 | 51,6 |
| 5 | 703,7 | 67,4 | 38,1 | 123,1 | 67,7 |
| 6 | 658,3 | 67,4 | 35,6 | 157,7 | 81,1 |
| 7 | 615,3 | 67,4 | 32,7 | 192,6 | 92,1 |
| 8 | 574,1 | 67,4 | 29,8 | 227,6 | 101,1 |
| 9 | 532,8 | 67,4 | 27,0 | 264,6 | 108,3 |
| 10 | 495,1 | 67,4 | 24,4 | 299,1 | 114,0 |
| 11 | 458,7 | 67,4 | 22,1 | 333,3 | 118,6 |
| 12 | 425,7 | 67,4 | 18,2 | 367,0 | 121,7 |
| 13 | 393,2 | 67,4 | 15,6 | 400,3 | 123,5 |
| 14 | 359,6 | 67,4 | 13,6 | 434,9 | 124,6 |
| 15 | 329,5 | 67,4 | 12,1 | 465,8 | 125,2 |
| 16 | 300,2 | 67,4 | 10,9 | 495,9 | 125,6 |
| 17 | 271,8 | 67,4 | 10,0 | 525,1 | 125,8 |
| 18 | 244,4 | 67,4 | 9,2 | 553,2 | 125,9 |
| 19 | 217,4 | 67,4 | 8,1 | 581,1 | 126,0 |
| 20 | 192,9 | 67,4 | 7,6 | 606,1 | 126,0 |
| 21 | 169,6 | 67,4 | 7,1 | 629,9 | 126,1 |
| 22 | 147,8 | 67,4 | 6,7 | 652,1 | 126,1 |
| 23 | 127,4 | 67,4 | 6,4 | 672,8 | 126,1 |
| 24 | 107,8 | 67,4 | 5,6 | 693,2 | 126,1 |
| 25 | 91,1 | 67,4 | 5,3 | 710,2 | 126,1 |
| 26 | 75,8 | 67,4 | 5,1 | 725,7 | 126,1 |
| 27 | 62,1 | 67,4 | 4,9 | 739,6 | 126,1 |
| 28 | 50,0 | 67,4 | 4,7 | 751,9 | 126,1 |
| 29 | 39,2 | 67,4 | 3,8 | 763,6 | 126,1 |
| 30 | 30,5 | 67,4 | 3,7 | 772,4 | 126,1 |
| 31 | 23,1 | 67,4 | 3,5 | 779,9 | 126,1 |
| 32 | 17,1 | 67,4 | 3,4 | 786,0 | 126,1 |
| 33 | 12,4 | 67,4 | 3,3 | 790,9 | 126,1 |
| 34 | 8,4 | 67,4 | 2,4 | 795,8 | 126,1 |
| 35 | 5,8 | 67,4 | 2,3 | 798,5 | 126,1 |
| 36 | 3,9 | 67,4 | 2,2 | 800,5 | 126,1 |
| 37 | 2,5 | 67,4 | 2,1 | 801,9 | 126,1 |
| 38 | 1,5 | 67,4 | 2,1 | 803,0 | 126,1 |
| 39 | 0,9 | 67,4 | 1,3 | 804,4 | 126,1 |
| 40 | 0,5 | 67,4 | 1,3 | 804,8 | 126,1 |
